# Acute Effects of Caffeine Supplementation on Physical Performance, Physiological Responses, Perceived Exertion, and Technical-Tactical Skills in Combat Sports: A Systematic Review and Meta-Analysis

**DOI:** 10.3390/nu14142996

**Published:** 2022-07-21

**Authors:** Slaheddine Delleli, Ibrahim Ouergui, Hamdi Messaoudi, Khaled Trabelsi, Achraf Ammar, Jordan M. Glenn, Hamdi Chtourou

**Affiliations:** 1 Research Unit, Physical Activity, Sport and Health, UR18JS01, National Observatory of Sport, Tunis 1003, Tunisia; sdelleli2018@gmail.com (S.D.); hamdimessaoudihamdi@gmail.com (H.M.); h_chtourou@yahoo.fr (H.C.); 2 High Institute of Sport and Physical Education of Sfax, University of Sfax, Sfax 3000, Tunisia; trabelsikhaled@gmail.com; 3 High Institute of Sport and Physical Education of Kef, University of Jendouba, Kef 7100, Tunisia; ouergui.brahim@yahoo.fr; 4 Research Laboratory: Education, Motricity, Sport and Health, EM2S, LR19JS01, University of Sfax, Sfax 3000, Tunisia; 5 Department of Training and Movement Science, Institute of Sport Science, Johannes Gutenberg-University Mainz, 55131 Mainz, Germany; 6 Research Laboratory, Molecular Bases of Human Pathology, LR19ES13, Faculty of Medicine, University of Sfax, Sfax 3000, Tunisia; 7 Interdisciplinary Laboratory in Neurosciences, Physiology and Psychology: Physical Activity, Health and Learning (LINP2), UPL, Paris Nanterre University, UFR STAPS, F-92000 Nanterre, France; 8 Department of Health, Exercise Science Research Center Human Performance and Recreation, University of Arkansas, Fayetteville, AR 72701, USA; jordan@neurotrack.com; 9 Neurotrack Technologies, 399 Bradford St., Redwood City, CA 94063, USA

**Keywords:** caffeine, supplementation, ergogenic aid, martial arts

## Abstract

Although the effects of caffeine supplementation on combat sports performance have been extensively investigated, there is currently no consensus regarding its ergogenic benefits.This systematic review with meta-analysis aimed to summarize the studies investigating the effects of caffeine supplementation on different aspects of performance in combat sports and to quantitatively analyze the results of these studies to better understand the ergogenic effect of caffeine on combat sports outcomes. A systematic search for randomized placebo-controlled studies investigating the effects of caffeine supplementation on combat sports’ performance was performed through Scopus, Pubmed, Web of Science and Cochrane Library databases up to 18 April 2022. Random-effects meta-analyses of standardized mean differences (Hedge’s g) were performed to analyze the data. Twenty-six studies of good and excellent methodological quality (based on the Pedro scale) fulfilled the inclusion criteria. The meta-analysis results revealed caffeine has a small but evident effect size (ES) on handgrip strength (ES = 0.28; 95% CI: 0.04 to 0.52; *p* = 0.02), and total number of throws during the special judo fitness test (SJFT) (ES = 0.42; 95% CI: 0.06 to 0.78; *p* = 0.02). Regarding the physiological responses, caffeine increased blood lactate concentration ([La]) in anaerobic exercise (ES = 1.23; 95% CI: 0.29 to 2.18; *p* = 0.01) and simulated combat (ES = 0.91; 95% CI: 0.34 to 1.47; *p* = 0.002). For Heart Rate (HR), caffeine increased HR final (ES = 0.31; 95% CI: 0.11 to 0.52; *p* = 0.003), and HR 1min (ES = 0.20; 95% CI 0.004 to 0.40; *p* = 0.045). However, caffeine had no impact on the countermovement jump height, the SJFT index, the judogi strength-endurance test, the number and duration of offensive actions, HR at the end of the fight, and the rating of perceived exertion. Caffeine supplementation may be ergogenic for a range of combat sports aspects involving isometric strength, anaerobic power, reaction time, and anaerobic metabolism. However, supplementation effects might be ineffective under certain circumstances, indicating supplementation needs to take into account the performance metric in question prior to creating a dosing protocol.

## 1. Introduction

Combat sports involving striking (e.g., karate, taekwondo, boxing, kickboxing) and grappling (e.g., judo, wrestling, jiu-jitsu) sports are both characterized by periods of high-intensity actions alternated with low-intensity periods [1,2]. Due to its intermittent nature, both grappling and striking sports require a substantial contribution of aerobic and anaerobic metabolism [3,4]. During a typical contest, combat sports athletes use a variety of offensive and defensive actions to overcome the opponent [5]. Thus, the success in these sports is determined by technical-tactical performance which is supported by multiple physical, physiological and psychological aspects [1].

In high-level competitive sports, performance is generally evenly distributed and an athlete’s likelihood of winning a competition is often decided by minor nuances [6]. In combat sports, the highly dynamic interaction between opponents requires a significant solicitation of the glycolytic system [4], increasing muscular acidosis (i.e., production of H^+^), disturbing the acid-base balance [7] and compromising performance [8]. In an attempt to delay the inhibitory effects induced by acidosis during combat, and ultimately enhance performance, competitive athletes regularly utilize dietary supplements as ergogenic aids [9]. Among accessible sports supplements, caffeine (1,3,7-trimethylxanthine) is one of the most consumed substances in the world of sport [10]. In fact, since the removal of caffeine from the World Anti-Doping Agency banned list, urinary caffeine concentration increased significantly from 2004 to 2015 with 76% of competitive athletes consuming caffeine to improve performance [11].

The ergogenic effects of caffeine have been observed in endurance activities, as well as in intermittent and strength activities [10,12,13,14,15,16]. The observed benefits effects of caffeine supplementation on sports performance have been explained by different mechanisms [17]. Specifically, in the central nervous system, caffeine acts as a potent adenosine receptor antagonist and inhibits the negative effects that adenosine induces on neurotransmission, excitation and pain perception, by blocking adenosine A_1_ and A_2a_ receptors [16,18,19,20]. Atthe peripheral level, caffeine was reported to act in enhancing muscle contraction throughout calcium ion (Ca^2+^) mobilization, increasing sodium/potassium (Na^+^/K^+^) pump activity to potentially enhance excitation-contraction coupling, thus inhibiting phosphodiesterase activity [17].

In a review including 21 meta-analyses, Grgic et al. [21] reported caffeine administration elicited ergogenic effects on muscle endurance, strength, anaerobic power and aerobic endurance. However, the effectiveness of caffeine as an ergogenic aid appears to be varied, with approximately 33% of individuals failing to improve performance after supplementation [22]. In this context, several factors have been reported to modulate the impact of caffeine supplementation, including dose, training status, ingestion time, time of day, sex, habituation, genetics, and exercise modality [12,21,22,23,24].

In combat sports, caffeine effects are inconsistent [8], which prevents drawing concretized conclusions on this topic. To date, several meta-analyses [19,24,25,26,27,28,29,30,31,32,33,34,35,36] investigating the impact of caffeine supplementation on sports performance have been conducted. In combat sports specifically, a recent systematic review with a meta-analytic approach [37] was conducted; however, the aforementioned meta-analysis was limited to some combat sports performances and neglected several other variables, such as the handgrip strength, the dynamic strength-endurance performance, the special judo fitness test index, the heart rate, and the rate of perceived exertion. Moreover, conducting a meta-analysis following the updated Preferred Reporting Items for Systematic Reviews and Meta-Analyses guidelines (PRISMA) is recommended to generate reliable and transparent findings [38], which was not the case in the aforementioned meta-analysis. Moreover, based on the work by Diaz-Lara et al. [37], it seems that choosing one dose in the case of multi-arms studies is an inappropriate method, as the research question is to compare the drug in all dosages to the placebo. This is evident, as all comparisons (also called relative effects or contrasts) to the placebo should be included in the same meta-analysis, according to the Cochrane handbook [39].

Given the common use of caffeine in combat sports and the lack of scientific consensus on its effects, a more thorough meta-analysis is required to resolve the inconsistencies. Thus, the goal of this paper was to conduct a systematic review with more accurate pooled effects sizes of studies that have investigated the effect of caffeine supplementation on performance outcomes in combat sports, and provide possible explanations for efficacy discrepancies, while also discussing practical applications that could help guide future supplementation protocols.

## 2. Methods

### 2.1. Eligibility Criteria

Inclusion criteria were applied following the PICOS (participants, intervention, comparators, outcomes, and study design) model. Articles were included if they were conducted on active combat sports athletes, using caffeine supplementation as an intervention in any form. The studies were retrieved if caffeine supplementation was controlled by a placebo intervention. Moreover, only the studies including performance measurement using combat simulation tasks and/or physical tests using a double-blind placebo-controlled design were included. These eligibility criteria resulted in the exclusion of studies that were conducted in non-combat sports athletes or did not include a caffeine supplementation protocol. In addition, articles were excluded if caffeine was administered in combination with other substances without a direct caffeine-placebo comparison. Trials that did not follow the double-blind placebo-controlled design were also excluded. Moreover, trials conducted during Ramadan were excluded as fasting can affect total sleep time [40], and induce dehydration [41], which could compromise performance. Furthermore, books, citations, conference proceedings, systematic reviews and narrative reviews were excluded.

### 2.2. Search Strategy

The systematic literature search was performed up through 18 April 2022 following the 2020 Preferred Reporting Items for Systematic Reviews and Meta-Analyses (PRISMA) [38] and the PRISMA guidelines adapted to sports science [42]. An a priori protocol was devised and can be requested from the corresponding author. PubMed, Scopus, Web of Science and Cochrane Library were searched without time limits or filters. The search syntax included the following keywords: Caffeine, coffee, combat sports, martial arts, judo, taekwondo, boxing, taekwondo, wrestling and jiu-jitsu. Appropriate Boolean connectors (AND, OR) were utilized to connect the various keywords and medical subject headings; (MeSH) terms were also used where appropriate. Combinations and search results on each database are presented in Table 1.

An additional search was performed by screening the reference lists of the included studies and related review papers using Google Scholar. Two authors performed the search independently. Any discrepancies between the authors in the study selection were resolved by consulting a third reviewer. No language restriction was applied in the search since this fact could introduce the risk of ignoring key data [42]. The search results were imported and treated using the software “Endnote 20” (Camelot UK Bidco Limited-Clarivate, London, UK).

### 2.3. Selection Process

The retrieved articles were firstly assessed for duplication using the software “Endnote 20” (Camelot UK Bidco Limited-Clarivate, London, UK) before being considered for inclusion. After duplicates were removed, a review of all relevant article titles was conducted before an examination of article abstracts and then full published articles. Two reviewers conducted the process independently. Consensus was used to resolve disagreements between the two reviewers. The reasons for the exclusion in each step were recorded.

### 2.4. Data Extraction and Coding

For all studies meeting the inclusion criteria, data were summarized in a spreadsheet using Microsoft Excel software. A piloted data extraction form with the following items: Author(s), year of publication, study design, sample size and combat sport name, timing after caffeine supplementation, caffeine dose (≤3: low; 4–6: moderate; >6: high), the form of caffeine supplement (capsule; chewing gum), the exercises used to assess performance and the main results was used. The extracted data were presented in Table 2. Moreover, the characteristics of participants in each study were extracted. Data reporting sex (males; females), age (≤18 years: adolescent; >18 years; adult athlete), experience (<5 years: amateur; ≥5 years: elite) of athletes, as well as their habitual caffeine intake were presented in Table 3. The mean and standard deviation were retrieved using WebPlot Digitizer (Pacifica, CA, USA, Version: 4.5) (https://automeris.io/WebPlotDigitizer, accessed on 28 April 2022) when data were displayed inside a graphic and no further data was provided upon request. To avoid any selection bias and data extraction flaws, the data extraction process was double-checked by a second reviewer [43]. Otherwise, if extraction was impossible (e.g., the figure was not clear or no figure was presented), the corresponding authors were contacted. If we did not receive an answer, the trial was excluded from the analysis.

### 2.5. Risk ofBias Assessment

The 11-item PEDro scale was used to assess the methodological quality of the included studies [70,71]. The quality of studies was assessed based on the study conducted by Mielgo-Ayuso et al. [72] as follows: excellent quality (9–10 points), good quality (6–8 points), fair quality (4–5 points), and poor quality (<4 points). Two authors assessed the methodological quality independently and checked the studies that already have a score, with discussion and consensus over any observed differences.

### 2.6. Data Synthesis and Analyses

Meta-analysis was conducted using the ComprehensiveMeta-Analysis software (version3; Biostat, Englewood, NJ, USA). To conduct meta-analysis, a minimum of three studies was requested. Standardized mean differences (Hedge’s g) and 95% confidence intervals (95% CI) were calculated between the placebo and caffeine trials using the variable mean and standard deviation values, the trial correlation, and the number of participants. Because no correlation values were published in any of the included investigations, a cautious 0.5 correlation was assumed across trials [73]. In the case of a multi-arms study (e.g., more than one caffeine dose), values were averaged across the different conditions [30]. The effects size (Hedge’s g) was interpreted as follows: Small (g < 0.50), medium (0.50 ≤ g < 0.80), and large (g ≥ 0.80) [74]. The inverse-variance random-effects model for meta-analysis was chosen since it assigns a proportionate weight to trials depending on the magnitude of their individual standard errors [75] and makes analysis easier while accounting for study heterogeneity [76]. This choice of weights minimizes the imprecision (uncertainty) of the pooled effect size [75]. To calculate heterogeneity among studies, the Q and I^2^ statistics were used. Heterogeneity was indicated if the Q statistic reached a significance of *p* < 0.05 and I^2^ > 50% [77]. The I^2^ statistic was classified as low (<50%), moderate (50–75%) and high (>75%) [34]. Because there was an insufficient number of studies to conduct subgroup or meta-regression analysis, the different moderators that could explain the observed heterogeneity (i.e., dose, timing, form, sample age, sample sex, expertise level, tolerance to caffeine, genetic background, time of day) were discussed in a qualitative way to avoid false positive or negative effect due to a lack of information rather than a smaller (or absent) effect [75]. To identify publication bias, funnel plots’ potential asymmetries, Begg and Mazumdar’s rank correlation test [78], and Egger’s linear regression test [79] were used. Moreover, the Duval and Tweedie’s trim and fill method [80] was used to identify missing studies. The stability of the pooled ES of each study was assessed using sensitivity analyses and involved removing individual studies from the analysis and computing the impact of theexcluded study [81]. In addition, a cumulative meta-analysis, which aims to aggregate accumulating evidence with additional studies based on their chronological order [82], was conducted to further ensure the stability and reliability of the results. When data were included in qualitative synthesis (i.e., not in meta-analysis), the percentage of improvement was calculated using the equation proposed by Lopez-Gonzalez et al. [8] [% improvement = (value with supplementation—value with placebo)/value with placebo × 100)]. A significance of *p* < 0.05 was adopted for all analyses.

## 3. Results

### 3.1. Studies Selection

**Figure 1:**
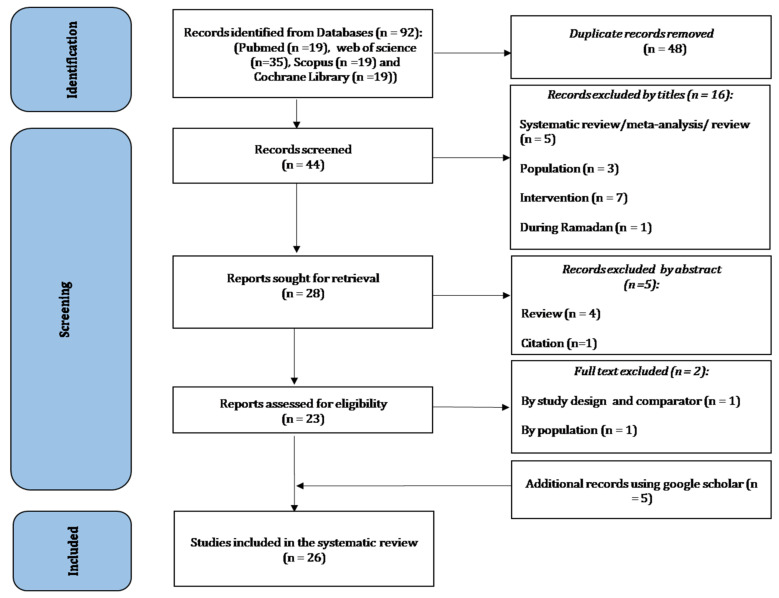
Flow diagram of the search process.

Figure 1 provides the flow diagram of the search process. A total of 92 records were found via the initial search. Among these, 48 were excluded as duplicates (40 using Endnote and eight manually), with 44 records remaining. Records’ titles were screened with 16 being removed. After screening the records’ abstracts for relevance, five wereremoved, and 23 reports remained and were assessed for eligibility. By applying the eligibility criteria, two full texts were excluded based on the study design (i.e., does not meet the double-blind placebo-controlled trial criterion) and population (see Table S1 for the list of excluded studies). After conducting an additional search, five articles were identified as potentially relevant studies, resulting in a total of 26 studies included in this systematic review.

### 3.2. Studies Characteristics

The 26 studies included in this systematic review regrouped 17 trials [44,45,46,47,48,49,50,51,52,53,54,55,56,57,58,59,60] from grappling, eighttrials [61,62,63,64,65,66,67,68] from striking combat sports, and one trial [69] from mixed martial arts (MMA).

Within the included studies, caffeine was administered based on the combat sport athletes’ body mass (i.e., relative dose). Using a capsule form, the caffeine doses used in the trials were 2 mg·kg^−1^ [65], 3 mg·kg^−1^ [48,49,50,57,59,60,68], 4 mg·kg^−1^ [45,54], 5 mg·kg^−1^ [44,46,47,53,56,61,63,65,66,67,69], 6 mg·kg^−1^ [48,50,53,56,57,60,62], 9 mg·kg^−1^ [50], and 10 mg·kg^−1^ [54]. In addition, both the repeated dose (5 × 2 mg·kg^−1^) and selective dose (i.e., dose given based on performance decrement in the first trials) of caffeine were used [54]. From the 26 included studies, two trials [58,63] examined the effect of caffeine and sodium bicarbonate separately and in combination. Using a capsule form, the timing between caffeine supplementation and the subsequent performance testing was 30 min [44,62], 45 min [54], 50 min [63], 60 min [45,46,47,48,49,50,52,53,55,56,58,59,60,61,63,64,65,66,67,68,69], and 120 min (i.e., testing after a training session) [47]. However, when using a caffeinated chewing gums form, only one study [51] investigated the effect of this supplementation form using both 2.7 mg·kg^−1^ and 5.4 mg·kg^−1^ doses 15 min before testing.

Since the objective of each study was to enhance performance as much as possible, from the 26 included studies, six [45,48,49,52,57,63] were multi-arm studies, with variation in dose [48,49,52,57,63] or in timing [47].

To reveal any effect of caffeine supplementation on physical performance, several testing procedures have been proposed. More specifically, in both grappling and striking combat sports, a set of non-specific physical tests were performed. For the upper body, the arm ergometer test [44], bench-press [48,59], and handgrip strength [44,46,47,51,57,62] were the most used tests. For the lower body, the leg press [65], vertical jump tests (i.e., Sergeant and countermovement jump (CMJ) tests) [46,47,57,58,62,63], the repeated sprint anaerobic test [65], maximal static lift [48,49], and the Wingate test [64] were used. However, to examine the effect of caffeine supplementation on specific performance, specific physical fitness tests were used to mimic the technical and physical aspects of real competition. The special judo fitness test (SJFT) [45,47,50,51,52,55,58] and the judogi grip strength test [44,51,57] were used to evaluate grappling performance, while the dollyo-chagui kick test [61,67], the taekwondo specific agility test (TSAT), the 10s frequency speed of kick test (FSKT-10s) and its multiple version (FSKT-mult) in taekwondo [68], the karate specific aerobic test (KSAT) [63], the repeated punching test in mixed martial arts (MMA) [69], and the Pittsburgh Wrestling Performance Test (PWPT) in freestyle wrestling [54]. However, to identify the variation of technical-tactical aspects regarding caffeine supplementation, simulated combats were performed in taekwondo [66,67], boxing [62], judo [44,48,49,54,60], wrestling [54] and Brazilian jiu-jitsu (BJJ) [49].

To investigate the associated physiological and psychological responses to caffeine supplementation, heart rate (HR) [44,45,47,50,51,53,54,55,62,63,66,67], blood lactate concentration ([La]) [44,46,47,49,51,52,53,54,58,63,64,66,67], rating of perceived exertion (RPE) [44,45,47,49,50,51,52,53,56,58,62,63,65,66,67,68,69], rating of perceived fatigue (RPF) [44,54], perceived recovery status (PRS) [56], and pain perception [47,65] were measured.

### 3.3. Population Characteristics

In the 26 reviewed studies, there were a total of 323 combat sport athletes recruited. From the grappling modality, 223 athletes were recruited with 124 from judo [45,46,47,50,51,52,53,55,56,58,59], 88 from jiu-jitsu [46,47,51,55,58,81], and 11 from freestyle wrestling [54]. From striking sports, 89 athletes were recruited, with 18 from karate [63,65], 53 from taekwondo [61,66,67,68], and 18 from boxing [62,64]. From MMA, 11 athletes were recruited [69].

Regarding the sample sex distribution, a major participation of male athletes was noticed. Sixteen studies recruited male athletes (i.e., 187 athletes), two studies (i.e., 23 athletes) [55,65] recruited female athletes, four studies [55,57,58,66] combined both sexes (i.e., 35 males and 33 females, respectively), and four studies [51,59,61,81] did not identify their sample sex (i.e., 45 athletes).

### 3.4. Risk of Bias Assessment

After checking the score of 10 studies that have been already scored in previous studies [8,24] and assessing the score of the remaining ones, the 26 included studies showed high methodological quality, with one study [47] receiving a score equal to 9 points and all other received 10 points. The study conducted by Carmo et al. [47] failed to satisfy the “less than 15% dropout” criterion since the data were obtained from less than 85% of subjects who were initially recruited. The Pedro scores of the included studies are presented in Table 2.

### 3.5. Meta-Analysis Results

#### 3.5.1. Effects of Caffeine Supplementation on Physical Performances

##### CMJ

**Figure 2:**
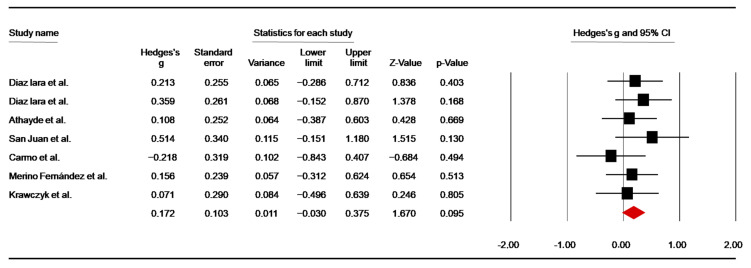
Forest plot of the effect of caffeine intake on CMJ [46,47,48,49,59,60,64].

Seven trials [46,47,48,49,59,60,64] investigated the effect of caffeine supplementation on CMJ height. To standardize testing circumstances and achieve a valid measurement of CMJ, the data were retrieved from pre-combat in two trials [46,49] and from pre-training (i.e., 60 min post-ingestion) in another one [47]. The pooled data from these investigations showed a non-significant ES of 0.17 (SE = 0.10; 95% CI: −0.03 to 0.38; z = 1.67; *p* = 0.095; Figure 2), without significant heterogeneity (Q = 3.24; df = 6; *p* = 0.78; I^2^ = 0%). A funnel plot (Figure S1) showed no evidence of publication bias, which was confirmed by Begg and Mazumdar’s rank correlation test and Egger’s linear regression test (Table S2). Moreover, Duval and Tweedie’s trim-and-fill test found no missing studies.

##### Handgrip Strength

**Figure 3:**
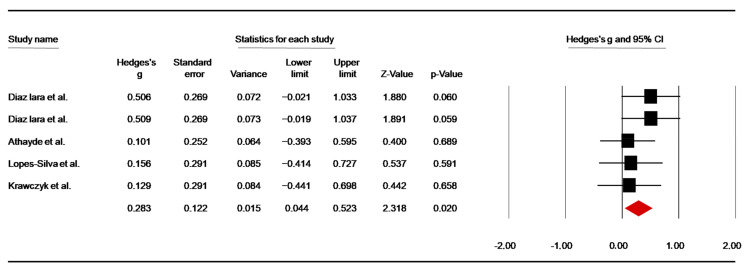
Forest plot of the total effect size of caffeine intake on handgrip strength [46,48,49,53,59].

Five studies [44,46,47,51,57] investigated the effect of caffeine supply on isometric handgrip strength. In two trials evaluating performance pre- and post-simulated combat [46,49], data were included from pre-combat. Pooled data showed a significant low ES of 0.28 (SE = 0.12; 95% CI: 0.04 to 0.52; z = 2.32; *p* = 0.02; Figure 3), without significant heterogeneity (Q = 2.38; df = 4; *p* = 0.67; I^2^ = 0.00%). A funnel plot (Figure S2) showed no evidence of publication bias, which was confirmed by Begg and Mazumdar’s rank correlation test and Egger’s linear regression test (Table S2). However, Duval and Tweedie’s trim-and-fill test identified one trimmed study with an adjusted point estimate of 0.22.

##### SJFT

Total Number of Throws

**Figure 4:**
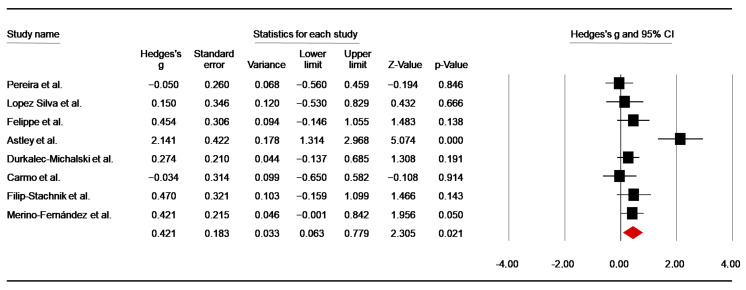
Forest plot of the effect of caffeine intake on SJFT number of throws [45,47,50,51,52,55,57,58].

Eight investigations [45,47,50,51,52,55,57,58] assessed the effect of caffeine supplementation on the total number of throws during the SJFT. From the study of Durkalec-Michalski et al. [50], the data of three conditions were averaged for analysis. From the study of Carmo et al. [47], pre-training data were included in the analysis, whereas in three studies using more than one SJFT [49,50,56], data from the first test (SJFT-1) were included. Pooled data from these studies showed a significant low ES of 0.42 (SE = 0.18; 95% CI: 0.06 to 0.78; z = 2.31; *p* = 0.02; Figure 4), with significant heterogeneity (Q = 22.71; *p* = 0.002; df = 7; I^2^ = 69.17%). A funnel plot (Figure S3) showed no evidence of publication bias, which was confirmed by Begg and Mazumdar’s rank correlation test and Egger’s linear regression test (Table S2). Moreover, Duval and Tweedie’s trim-and-fill test did not identify any missing study to the left of the mean.

SJFT-Index

**Figure 5:**
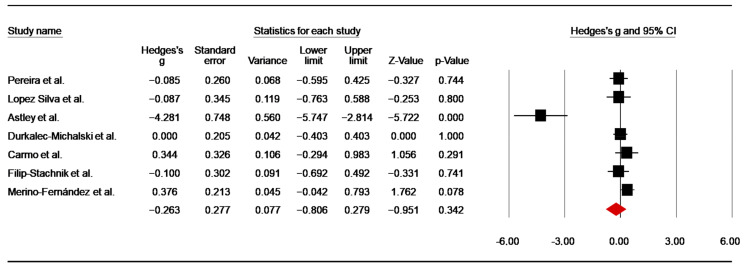
Forest plot of the total effect size of caffeine intake on SJFT index [45,47,50,51,52,55,57].

Seven investigations [45,47,50,51,52,55,57] assessed the effect of caffeine supplementation on SFT index. As mentioned above, data of the three conditions in the study of Durkalec-Michalski et al. [50] were averaged for analysis. From the study of Carmo et al. [47], pre-training data were included in the analysis, whereas in two studies using more than one SJFT [51,52], data from the first test (SJFT-1) were included. The pooled data showed a low ES of—0.26 (SE = 0.28; 95% CI: −0.81 to 0.28; z = −0.95; *p* = 0.34; Figure 5), with significant heterogeneity (Q = 37.23; df = 6; *p* < 0.001; I^2^= 83.89%). A funnel plot (Figure S4) showed evidence of publication bias, which was confirmed by Egger’s linear regression test. Moreover, Duval and Tweedie’s trim-and-fill test identified two trimmed studies, with an adjusted point estimate of −0.40. However, Begg and Mazumdar’s rank correlation test did not identify a significant risk of publication bias (Table S2).

##### Judogi Strength-Endurance Test

**Figure 6:**
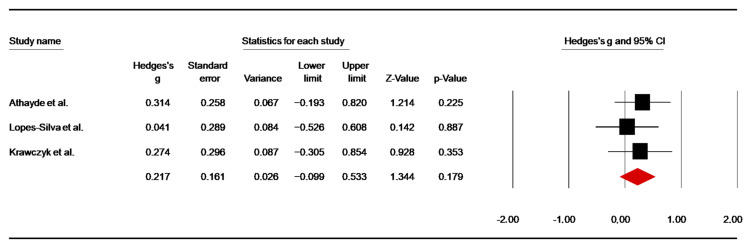
Forest plot of the effect of caffeine intake on judogi strength endurance test [46,53,59].

Three studies [44,51,57] investigated the acute effects of caffeine supplementation on the judogi dynamic strength endurance test. To standardize the condition, data of pre-match 1 from the study of Athayde et al. [46] and data of the first bout from the study of Lopes-Silva et al. [53] were included. The pooled data showed a low ES of 0.22 (SE = 0.16; 95% CI: −0.10 to 0.53; z = 1.34; *p* = 0.18; Figure 6), with no significant heterogeneity (Q = 0.55; df = 2; *p* = 0.76; I^2^= 0.00%). A funnel plot (Figure S5), showed no evidence of publication bias, which was confirmed by Begg and Mazumdar’s rank correlation test and Egger’s linear regression test (Table S2). Moreover, Duval and Tweedie’s trim-and-fill test did not identify any missing study to the left of the mean.

#### 3.5.2. Effects of Caffeine Supplementation on the Physiological Responses

##### Effects of Caffeine Supplementation on Blood Lactate

Post-Anaerobic Performance

**Figure 7:**
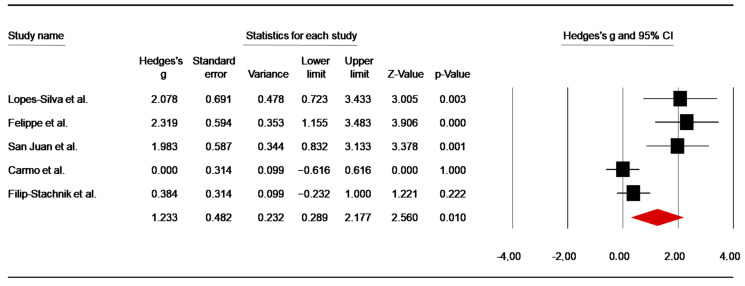
Forest plot of the effect of caffeine intake on [La] post-anaerobic exercise [47,51,52,58,64].

Five studies [45,49,50,56,62] assessed the effect of caffeine supplementation on [La] post-anaerobic exercise. Pooled data of these investigations showed a high ES of 1.23 (SE = 0.48; 95% CI: 0.29 to 2.18; z = 2.56; *p* =0.01; Figure 7), with significant heterogeneity (Q = 22.13; df = 4; *p* < 0.001; I^2^ = 81.92%). A funnel plot (Figure S6) showed an evidence of publication bias, which was confirmed by Egger’s linear regression test (*p* = 0.005). However, Begg and Mazumdar’s rank correlation test did not identify a risk of publication bias (Table S2). Moreover, Duval and Tweedie’s trim-and-fill test did not identify any missing study to the left of the mean.

Post-Simulated Combat

**Figure 8:**
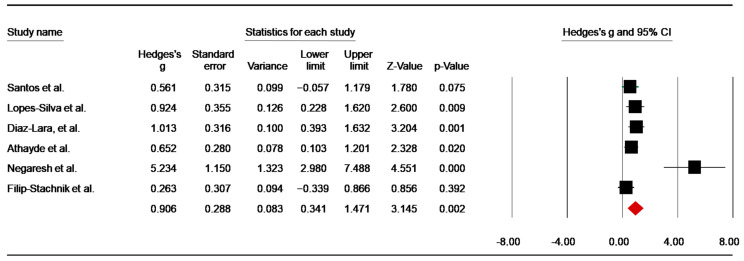
Forest plot of the effect of caffeine intake on [La] post-combat [46,49,51,54,66,67].

Six studies [44,47,49,52,64,65] assessed the effect of caffeine intake on [La] post-combat. The pooled data of these trials showed a significant high ES of 0.91 (SE = 0.29; 95% CI: 0.34 to 1.47; z = 3.15; *p* = 0.002; Figure 8), with significant heterogeneity (Q = 19.11; df = 5; *p* = 0.002; I^2^ = 73.83%). A funnel plot (Figure S7) showed evidence of publication bias, which was confirmed by Egger’s linear regression test (*p* = 0.01). However, Begg and Mazumdar’s rank correlation test did not identify a risk of publication bias (Table S2). Moreover, Duval and Tweedie’s trim-and-fill test did not identify a missing study to the left of the mean.

##### Effects of Caffeine Supplementation on Heart Rate Responses

HR Final

**Figure 9:**
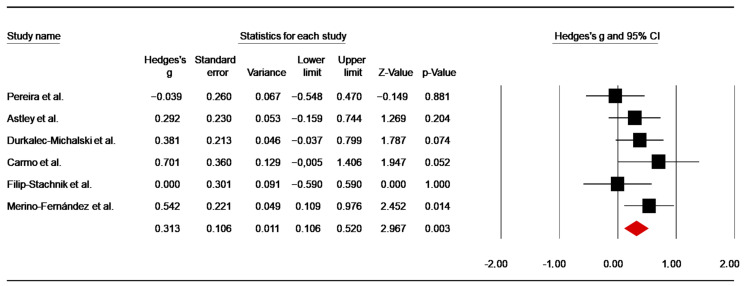
Forest plot of the effect of caffeine intake on HR final [45,47,50,51,55,57].

Six studies [45,48,49,53,55,82] investigated the effect of caffeine supplementation on HR final (i.e., HR measured immediately after the SJFT). From the study of Carmo et al. [47], data of pre-training were retrieved, whereas in the study of Durkalec-Michalski et al. [50] data of three caffeine conditions were averaged. The pooled data from these investigations showed a low ES of 0.31 (SE = 0.11; 95% CI: 0.11 to 0.52; z = 2.97; *p* = 0.003; Figure 9), without significant heterogeneity (Q = 5.26; df = 5; *p* = 0.39; I^2^ = 4.93%). A funnel plot (Figure S8) showed no evidence of publication bias, which was confirmed by Begg and Mazumdar’s rank correlation test and Egger’s linear regression test (Table S2). Moreover, Duval and Tweedie’s trim-and-fill test did not identify a missing study to the left of the mean.

HR 1 min

**Figure 10:**
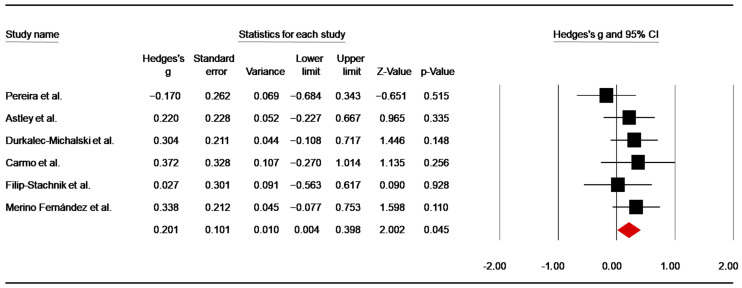
Forest plot of the effect of caffeine intake on HR 1min [45,47,50,51,55,57].

Six studies [45,48,49,53,55,82] assessed the effect of caffeine intake on HR 1 min after SJFT. The pooled data from these investigations showed a significant trivial ES of 0.20 (SE = 0.10; 95% CI 0.004 to 0.40; z = 2.00; *p* = 0.045; Figure 10), with no heterogeneity (Q = 3.29; *p* = 0.66; df = 5; I^2^ = 0.00%). A funnel plot (Figure S9) showed no evidence of publication bias, which was confirmed by Begg and Mazumdar’s rank correlation test and Egger’s linear regression test (Table S2). Moreover, Duval and Tweedie’s trim-and-fill test did not identify a missing study to the left of the mean.

HR Post-Combat

**Figure 11:**
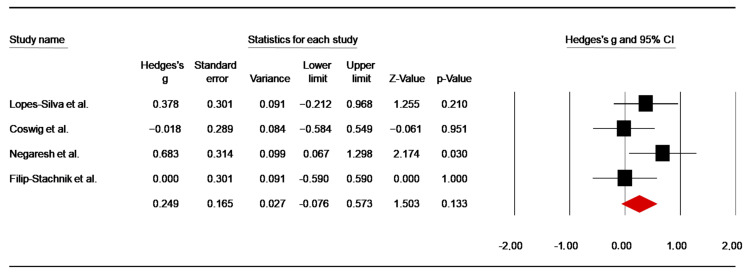
Forest plot of the effect of caffeine intake on end-of-fight HR [51,54,62,66].

Four studies [49,52,60,64] investigated the effect of caffeine supplementation on HR at the end of the fight. Data from the caffeine conditions reported in the study of Negaresh et al. [54] were averaged for the analysis. The pooled data from these investigations showed a low ES of 0.25 (SE = 0.17; 95% CI: −0.08 to 0.57; z = 1.50; *p* = 0.13; Figure 11), without significant heterogeneity (Q = 3.62; *p* = 0.31; df = 3; I^2^ = 17.21%). A funnel plot (Figure S10) showed no evidence of publication bias, which was confirmed by Begg and Mazumdar’s rank correlation test and Egger’s linear regression test (Table S2). Moreover, Duval and Tweedie’s trim-and-fill test did not identify any missing study.

#### 3.5.3. Effects of Caffeine Supplementation on RPE

##### Post-Anaerobic Exercise

**Figure 12:**
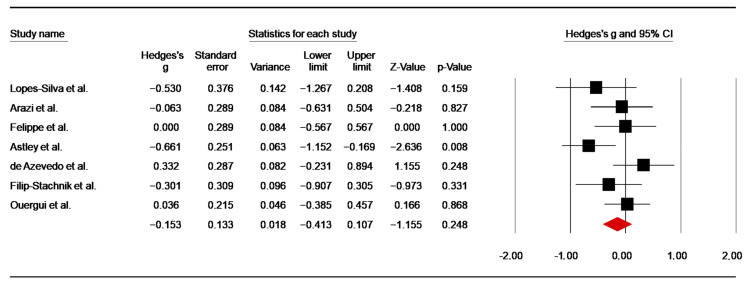
Forest plot of the total effect size of caffeine intake on RPE post-anaerobic exercise [45,51,52,58,65,68,69].

Seven studies [49,50,56,63,66,67,82] have investigated the effect of caffeine supplementation on RPE post-anaerobic exercise. For the studies using more than one test repetition (SJFT) [49,50,56], only the values from post-SJFT−1 were included in the analysis. In the study of Arazi et al. [65], the data from post-repeated anaerobic sprint tests were included in the analysis with values from low (i.e., 2 mg/kg) and moderate doses (i.e., 5 mg/kg) averaged. The pooled data from these studies showed a trivial ES of −0.15 (SE = 0.13; 95% CI: −0.41 to 0.11; z = −1.16; *p* = 0.25; Figure 12), without a significant heterogeneity (Q = 9.33; *p* = 0.16; df = 6; I^2^ = 35.67%). A funnel plot (Figure S11) showed no evidence of publication bias, which was confirmed by Begg and Mazumdar’s rank correlation test and Egger’s linear regression test (Table S2). Moreover, Duval and Tweedie’s trim-and-fill test did not identify a missing study to the left of the mean.

##### Post-Simulated Combats

**Figure 13:**
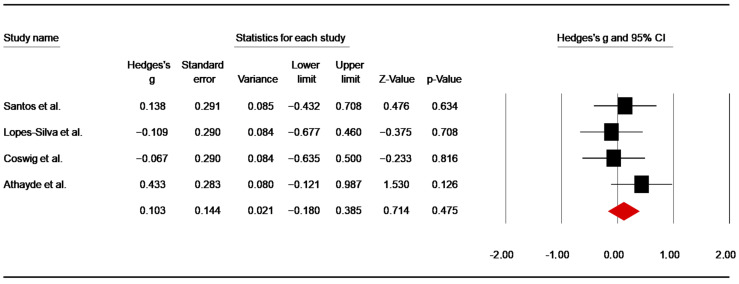
Forest plot of the effect of caffeine on RPE post-combat [56,62,66,67].

Four studies [54,60,64,65] assessed the effect of caffeine intake on RPE post-combats. From the studies using more than one combat [56,67], data after the first combat was included in the analysis. The pooled data from these investigations showed a non-significant low ES of 0.10 (SE = 0.144; 95% CI: −0.18 to 0.38; z = 0.714; *p* = 0.475; Figure 13), without significant heterogeneity (Q = 2.29; df= 3; *p* = 0.51; I^2^ = 0%). A funnel plot (Figure S12) showed no evidence of publication bias, which was confirmed by Begg and Mazumdar’s rank correlation test and Egger’s linear regression test (Table S2). Duval and Tweedie’s trim-and-fill test did not identify any missing study to the left of the mean.

#### 3.5.4. Effects of Caffeine Supplementation on Technical Skills

##### Number of Offensive Actions

**Figure 14:**
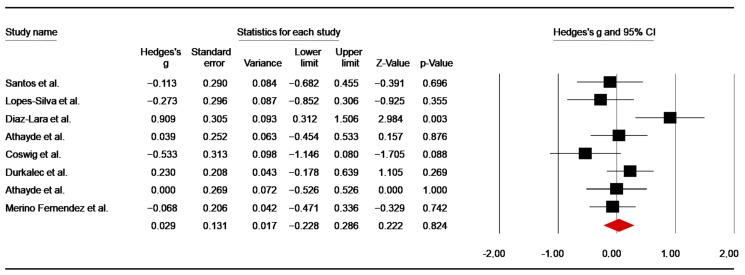
Forest plot of the effect of caffeine intake on number of offensives actions during combat [46,49,50,56,57,62,66,67].

Using simulated combats, eight studies [44,47,48,54,55,60,64,65] assessed the effect of caffeine intake on the number of offensive actions. From the study conducted by Durkalec-Michalski et al. [50], data of three conditions were averaged. For studies using more than one combat [44,47,54,65], data from the first combat were included in the analysis. The pooled data from these investigations showed a trivial ES of 0.03 (SE = 0.13; 95% CI: −0.23 to 0.29; z = 0.22; *p* = 0.82; Figure 14), with significant heterogeneity (Q = 14.03; *p* = 0.05; df = 7; I^2^= 50.10%). A funnel plot (Figure S13) showed no evidence of publication bias, which was confirmed by Begg and Mazumdar’s rank correlation test and Egger’s linear regression (Table S2). Moreover, Duval and Tweedie’s trim-and-fill test did not identify any missing study.

##### Duration of Offensive Actions

**Figure 15:**
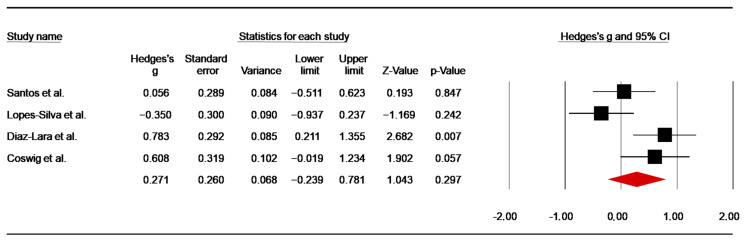
Forest plot of the effect of caffeine intake on duration of offensives actions during combat [49,62,66,67].

Four investigations [47,60,64,65] assessed the effect of caffeine supplementation on offensive actions’ duration. From the studies using more than one combat [49,67], data fromthe first combat were retrieved for analysis. The pooled data from these investigations showed a low ES of 0.27 (SE = 0.26; 95% CI −0.24 to 0.78; z = 1.04; *p* = 0.30; Figure 15), with significant heterogeneity (Q = 9.04; *p* = 0.03; df = 3; I^2^ = 66.80%). A funnel plot (Figure S14) showed no evidence of publication bias, which was confirmed by Begg and Mazumdar’s rank correlation test and Egger’s linear regression test (Table S2). Moreover, Duval and Tweedie’s trim-and-fill test did not identify any missing study.

#### 3.5.5. Stability and Reliability of The Results

Results of the sensitivity and cumulative meta-analysis are presented in the supplementary file (Figures S15–S42). Results of the leave-one-out sensitivity analysis indicated that the effect of caffeine supplementation on all of the above-mentioned pooled effects is robust and not significantly driven by any single study.For the cumulative meta-analysis, no significant fluctuation was found in the results of all the above-mentioned pooled effects, indicating the stability of the results over time.

## 4. Discussion

The present systematic review and meta-analysis aimed to summarize the available scientific studies investigating the effects of caffeine supplementation on combat sports’ performance and provide insight into the observed contradictions amongst different intervention protocols. The following parameters have been clustered for a more comprehensive assessment due to the differences in the outcomes evaluated in these studies.

### 4.1. Effects of Caffeine Supplementation on Physical Performance

#### 4.1.1. CMJ

The findings of the current meta-analysis were consistent with those reported previously in soccer players [83], revealing no significant impact of caffeine ingestion on jump height. However, it contradicts gains in vertical jump height described in another meta-analysis [24], where caffeine ingestion resulted in a positive ES of 0.17 for both trained and untrained participants. Moreover, caffeine supplementation resulted in a substantial ES ranging from 0.19 to 0.20 in team sports [29,35]. Although the present meta-analysis had comparable ES to the aforementioned studies, there was no statistically significant difference between caffeine and placebo. The discrepancy across results could be related to distinction in sports modalities [8] and the training levels [13]. However, even with combat sports athletes, Diaz-Lara et al. [37] found a significant effect of caffeine supplementation on jump height (Standardized Mean Difference (SMD) = 0.38). The discrepancy between our results and theirs could be related to the methods followed (e.g., adjusted effect size vs. standardized mean difference, and pooling data from multi-arms studies vs. using the dose with the greatest ergogenic effect). When accounting for exercise duration, caffeine is more effective in long-duration exercise than shorter duration exercise [84]. Specifically, caffeine appears to improve exercise performance lasting 45 s to 8 min [26]. Therefore, the lack of significant improvement in CMJ could be related to its short duration. However, this should be verified in future investigations.

#### 4.1.2. Handgrip Strength

Caffeine has been reported as a useful ergogenic aid for achieving an acute increase in maximal upper body strength [24] and in the present meta-analysis, caffeine supplementation improved handgrip strength performance. This supported the findings from a previous meta-analysis [36], which showed, independently of dose or form, supplementation increases handgrip strength. Regarding the ES, the present meta-analysis showed a higher impact than the aforementioned study. This could be related to sport specificity, as the ability to grip in grappling combat sports is of great importance to pull or push the opponent [85]. However, the ES recorded in the present meta-analysis was lower compared to that reported in female team sports [29]. This may be due to the fact that the comparable meta-analysis used a standardized mean difference, while in our meta-analysis an adjusted effect size was used. The improvement in isometric strength is based on several neurological characteristics including motor unit recruitment, motor unit synchronization, rate coding, and neuromuscular inhibition [86].

#### 4.1.3. SJFT

The SJFT was the most used physical fitness test to detect the possible effects of caffeine supplementation on specific performance in grappling combat sports. According to some investigations [45,53], the specificity of the testing procedure is an effective factor that may modulate the ergogenic potential effect of caffeine. This is reasonable as specific exercises are more preferred and motivating for different athletes [87]. The present meta-analysis revealed that caffeine supplementation improved the total number of throws during the test. This result supports the previous one reported by Diaz-Lara et al. [37] (SMD = 0.62), despite the distinction in the methodology used (as mentioned above). Moreover, as a measure of anaerobic performance, the increase in performance during the SJFT supports a previous meta-analysis [30], showing increased peak power and mean power output during the Wingate test, following supplementation. The most commonly accepted mechanism for the improvement in strength and anaerobic power is the influx of Ca2^+^ from the sarcoplasmic reticulum that promotes the creation of cross-bridges, therefore, increasing muscular power [88].

#### 4.1.4. SJFT Index

The SJFT index is a ratio between HR (i.e., HR _final_ and HR _1min_) and the total number of throws performed during the SJFT test. Consequently, its variation is dependent on the variation of its determinants [45]. As an ergogenic aid, it is commonly accepted that caffeine is efficient in delaying the onset of fatigue [46]. In combat sports, however, the results are inconsistent [8]. The present meta-analysis revealed there was no significant impact of caffeine on the SJFT index. The high heterogeneity across studies could inform us about the existence of some moderators that could modulate the ergogenic effects of caffeine. For instance, among the seven studies assessing the effects of caffeine supplementation on the SJFT index, one study by Lopes-Silva et al. [52] was conducted after a 5-day weight-loss period, which may have interfered with caffeine’s ergogenic effects [52]. This may be because the weight-loss period generated an imbalance in hormonal and hydroelectric homeostasis, inducing cardiovascular stress, altering immune function and depleting energy stores [8]. Moreover, the number of participants recruited in this study [52] was relatively small (i.e., six participants), with a high heterogeneity as they were divided into four weight categories; this could be considered a limiting factor because caffeine intake tends to present different individual responses [12]. Thus, it was recommended that caffeine supplementation must be used in a healthy, normal weight condition. Nevertheless, the observed decrease in the SJFT index seems to be relevant since an improvement of around 0.6% is sufficient to make a difference in elite-level sports [89]. The decrease in the fatigue level was generally attributed to an improvement in neuromuscular efficiency in the active muscle, which can be explained by the effect of caffeine in enhancing intra- and inter-muscle coordination [90] and the Na^+^/K^+^ATPase activity [88].

#### 4.1.5. Strength-Endurance Performance

An upper body test for grappling athletes [91], the judogi dynamic strength-endurance test, was used in only three investigations [44,51,57], with contradictory results. However, the meta-analysis showed caffeine supplementation did not affect the number of repetitions during the judogi dynamic strength-endurance test. Such a result was contradictory to what was expected based on a previous meta-analysis [27] using the bench press exercise. Regarding body parts, there are conflicting results about the effects of caffeine on upper- and lower-body musculature. Some studies suggested caffeine ingestion enhances one repetition-maximum (1RM) strength in the upper, but not lower-body musculature [24,27]. In contrast, it is suggested that larger muscles, such as those of the lower body, have a greater motor unit recruitment capability with caffeine intake than smaller muscles [25]. In combats sports, it seems that even with the same testing procedure (i.e., same muscle groups) there have been inconsistent results. This fact may indicate that other factors, such as the training phase (i.e., preparatory or competitive) can modulate caffeine effects. This is reasonable as the study conducted during the preparatory phase [46] did not show improvement, while those conducted in the competitive phase [53,59] reported performance enhancement in response to caffeine supplementation, even though the same dose and timing were provided.

#### 4.1.6. Reaction Time, Kick Speed and Agility

The effect of caffeine supplementation on reaction time was tested in two studies [61,67] involving striking combat sports. In those studies, it appeared that 5 mg·kg^−1^ of caffeine ingestion 1 h prior to testing was an effective strategy to enhance reaction time in male taekwondo athletes [61,67], with improvement ranging from 11.9% to 29%. This improvement was mainly located in the rectus femoris muscle [61], which is the most solicited muscle in the dollyo-chagui technique execution [92]. The enhancement in the intramuscular activity via potentiating neurotransmitter release and improving motor neuron transmission may explain these findings [93]. Due to the insufficient number of studies, a meta-analysis was not performed at this time. However, in a previous meta-analysis [83] on soccer players, it was reported that caffeine supplementation did not affect reaction time. This contradiction maybe related to the limited number of studies (i.e., twostudies) included in the aforementioned meta-analysis, thus affecting its validity. Regarding speed and agility, Raya-Gonzalez et al. [34] reported that caffeine has the most pronounced performance-enhancing effects on movement velocity with a pooled effect size ranging from 0.41 to 0.82. Moreover, Salinero et al. [35] found that a moderate dose of caffeine reduced the time to complete agility tests (0.41). Among the included studies, only Ouergui et al. [68] tested the impact of a low caffeine dose (i.e., 3 mg·kg^−1^) on specific agility performance in young taekwondo athletes. This investigation revealed caffeine resulted in lower TSAT time in comparison with placebo. Moreover, Ouergui et al. [68] reported the dose of caffeine increased the frequency speed of the kick test by 4%. Because speed, agility, and reaction time are vital performance determinants in combat sports, further investigations ofthese variables are required.

### 4.2. Effects of Caffeine on Physiological Responses

#### 4.2.1. Effects of Caffeine on Blood Lactate

The pooled effects showed a significant increase in [La] during simulated combat and high-intensity anaerobic performance. Since caffeine may increase the intensity of combat, a higher [La] concentration is a result of increased glycolytic anaerobic metabolism [56,66]. In this context, it was suggested that caffeine supplementation could be an effective pharmacological strategy to improve anaerobic capacity in combat sports [66]. These findings supported a previous meta-analysis [28], which reported a raw mean difference of +0.69 mmol·L^−1^ in [La] between caffeine and placebo in response to sub-maximal exercise (i.e., constrained to 60–85% maximal rate of oxygen consumption). Moreover, our results support previous findings in combat sports [37], showing an increase in [La] post- the first bout (SMD = 1.17), the second bout (SMD = 1.25), and the thirdbout (SMD = 1.98) of simulated combat. Therefore, it is clear that, independently of the caffeine doses or timing between supplementation and the subsequent performance, caffeine ingestion increased [La] in an intermittent high-intensity specific task [44,47,50]. However, the increased values of [La] following caffeine supplementation could be also related to an impairment of clearance, rather than to an increase in lactate production (i.e., via increased glycolysis activation) [28]. A high lactate level is associated with a high concentration of H^+^ ions (i.e., low pH), which can impair muscle performance through a reduction of energy supply via glycolysis and glycogenolysis [94]. In this case, the increase in [La] following caffeine supplementation without improving performance in some studies [45,64,81], could be related to an impairment of [La] clearance rather than the enhancement of the glycolytic metabolism via increased activity of the enzyme phosphofructokinase or stimulation by catecholamines [17,95]. These suggestions were explicitly reported by Rezaei et al. [63], who assumed that H^+^ buffering in blood resulted in an inhibitory effect on glycogen phosphorylase and phosphofructokinase enzymes. However, this suggestion seems to be restricted as buffering would not result in a potential reduced inhibitory effect. Another possible reason for the lack of [La] increase following intermittent exercise is the nature of the exercise. The work and recovery duration play a crucial role in caffeine’s ergogenic effects on intermittent exercise [96]. As a result, it was suggested that a sufficient recovery period seems to be effective at replenishing phosphocreatine stores and, consequently, maintaining the energy supply via the anaerobic alactic system [96].

#### 4.2.2. Effects of Caffeine on Heart Rate

The present meta-analysis showed caffeine supplementation was effective at increasing HR final and HR 1 min after the SJFT, but not HR at the end of the simulated combats. These findings are in line with previous results [28,32] showing no significant impact of caffeine on HR during submaximal exercise. In this regard, the effect of caffeine on HR was considered exercise intensity-dependent [28]. In fact, the impact of caffeine appears to decline as exercise intensity increases. This is likely due to the lack of HR difference post-combat and could be related to mental stress, which was superimposed on the caffeine impact [97]. The increased HR values before the effort could be related to mental stress anticipation [98], whereas the results were near to HR_max_ at post-round [62]. Since caffeine is an adenosine antagonist [16,18,19], the primary mechanisms involved in the action of caffeine on HR regulation are the activation of the sympathetic nervous system by the release of catecholamines from the adrenal medulla and corticosteroids from the adrenal cortex [99], and by the inhibition of phosphodiesterase enzymes [100]. Regarding HR recovery, it was reported that caffeine delayed parasympathetic regulation of heart rhythm following exercise, slowing HR recovery [101]. In this context, results from BJJ practitioners, showed not only increased peak HR and early HR recovery but also higher late HR recovery in the caffeine trial (5 mg·kg^−1^), as compared to placebo [44]. According to the included trials, the impact of caffeine on HR responses seems to be dose-dependent, as the HR increase was relative to the ingested doses [50].

### 4.3. Effects of Caffeine on RPE

Caffeine ingestion has been reported to modify perceived exertion (RPE), which can result in altered performance [19]. In the present meta-analysis, there were no significant impacts of caffeine supplementation on RPE post-anaerobic exercise and simulated combats. These findings supported those reported in previous meta-analyses [27,29,32], showing that caffeine did not affect RPE. However, these results contradict those reported by Glaister and Gissane [28] and Grgic and Mikulic [102], who revealed caffeine supplementation has a suppressive effect on RPE following submaximal exercise and a 1RM barbell back squat performance. Moreover, it was reported that the RPE decrease explains up to 29% of the ergogenic effect of caffeine on sub-maximal aerobic exercise performance [19]. Since the reduction in blood flow to the brain during exercise has been identified as a factor promoting fatigue [103], the previously observed effects of caffeine supplementation on RPE are mediated by caffeine’s impact on cerebral oxygenation [89] via crossing the blood-brain barrier [104] and inhibiting the A_1_ and A_2a_ adenosine receptors [16,18,19]. Nevertheless, the discrepancy across studies could be related to several factors including the variation in the testing procedure and the participant profiles. It has been theorized that exercise complexity may affect the amount of fatigue, given that complex, multi-joint exercises stimulate more muscle groups and, hence, demand higher exertion [105]. Under simulated combat conditions, caffeine served to increase combat load, while fatigue perception was unaffected. Thus, it seems possible to confirm caffeine may have the capacity to enhance the physical performance of combat sports athletes without producing higher values of fatigue. The increased time to fatigue may be attributed to the reduction of the central fatigue rate [106]. Moreover, because the specific testing procedures are most similar to competition and mimic the demands of training and in-competition sessions, athletes were familiarized and there was no effect of learning that could increase fatigue levels. Additionally, given that the RPE scale was created to reflect both the heart rate response to exercise and the level of exertion [107], it is perhaps not surprising to find any difference in RPE, as HR post-combats did not appear to be altered by caffeine. Furthermore, the lack of RPE variation in response to high-intensity exercise could be related to other extrinsic factors (i.e., timing of evaluation, athlete profile, time of day, familiarity with the testing procedure).

It should be noted that the use of scales to quantify RPE has been criticized in numerous ways [27]. For instance, Davis and Green [17] demonstrated RPE measures can be coarse in detecting perceptual responses during high-intensity exercise. In addition, Gergic et al. [105] attributed the lack of difference in RPE following caffeine ingestion to the timing of evaluation as, in high-intensity exercise, RPE is evaluated almost exclusively at exercise termination. In combat sports, the high-intensity efforts are intercepted by low-intensity periods and do not follow a constant load pattern, which could explain the lack of caffeine effect on RPE found in the present meta-analysis. This may be evident, as caffeine ingestion reduced RPE recorded during exercise, but does not affect RPE obtained at the end of exhausting exercise [19]. Caffeine appears to delay autonomic recovery following exercise [108], as throughout judo matches, RPE increased and RPR decreased independent of performance enhancement [56]. All studies included in our meta-analysis only included an RPE assessment at the end of the test or combat, but it remains interesting for future studies to consider RPE throughout exercise.

### 4.4. Effects of Caffeine on Technical-Tactical Aspects

The present meta-analysis revealed caffeine supplementation did not improve the number or duration of attacks. This result was surprising as caffeine ingestion has been associated with anincrease in anxiety arousal, energy, impulsiveness, and risk-taking [65,107,108], which could affect high-intensity offensive activities. Our findings were partially (i.e., only for the number of offensives actions) in line with those reported in a previous meta-analysis [37], showing caffeine supplementation did not affect the number of offensive actions during the first combat. However, the previous meta-analysis reported caffeine improved the number of offensive actions in the second combat (SMD = 0.40) and the duration of offensive actions (SMD = 0.58). As we selected data only from the first combat, the discrepancy between our results and those of Diaz-Lara et al. [37] concerning the effect of caffeine supplementation on the duration of offensive actions may be related to the inclusion of data from the first and the second combat in their meta-analysis. The lack of performance enhancement after caffeine supplementation and the high heterogeneity across studies maybe related to the divergence in combat duration and modality (i.e., striking vs. grappling). In fact, it was suggested that match-derived judo performance variables were not sensitive enough to distinguish between small or medium effects of caffeine [46,56]. This has been explained by the fact that judo combat is composed by acyclic actions and is dependent on the opponent’s level and experience and his/her technical-tactical strategies used during the fight [109]. Moreover, it seems that combat duration and level play a crucial role. In fact, a low dose (i.e., 3 mg·kg^−1^) of caffeine was sufficient to increase the number of offensive actions by 28% in 8 min of Brazilian jiu-jitsu simulated combat [49]. This increase in high-intensity actions was linked to a trend toward greater advantages, scoring actions, and submissions [49]. Furthermore, in male taekwondo athletes, Santos et al. [67] reported that 5 mg·kg^−1^ of caffeine 1 h before two simulated taekwondo combats did not increase the number of attacks in the first combat; however, a significant improvement of 37% was recorded in the second combat. Contrarily, using the same dose and respecting the same timing after supplementation, Lopes-Silva et al. [66] did not report time-motion variables’ improvement during simulated taekwondo combat. These contradictory results were explained by the fact that combat in the study conducted by Santos et al. [67] was performed between supplemented athletes (i.e., caffeine versus caffeine), while Lopes-Silva et al. [66] used supplemented athletes confronting non-supplemented ones. Through this, it was suggested that during simulated taekwondo combat, it is difficult to observe any performance variables’ improvement when caffeine is supplemented from one side (i.e., only one opponent receives caffeine) [66]. This result may be explained by the fact that in a 1 vs. 1 competitive situation, combat athletes’ behavior is directly influenced and constrained by that of the opponent [5]. Moreover, despite similarities between combat sports (i.e., intermittence), there are considerable differences between grappling and striking in terms of the technical-tactical characteristics and the effort-to-pause ratio [1,63]. For example, grappling combat sports require strength-endurance and power to perform grappling and throwing techniques [4]. However, striking combat sports are characterized by high-speed attack and defense movements to perform kicking and punching techniques [3,110]. These differences could explain, in part, the contradictory results between the two modalities, even when the same dose and timing after supplementation were provided (i.e., [67] vs. [46,56]). Therefore, and as the effort to pause ratio is modality-dependent [1], results from striking cannot be transferred to grappling contests, particularly, for technical-tactical skills.

### 4.5. Possible Moderating Factors

When conducting caffeine supplementation studies, inter-individual variability (i.e., sex, age, habitual caffeine consumption and training status) and supplementation protocols (i.e., dose, timing, form and time of day of measurement) should be taken into consideration.

#### 4.5.1. Effect of Sex

Sex has been identified as an important determinant of athletic performance [72]. In the context of caffeine supplementation, it has been reported that, with the same amount of caffeine, males can produce higher power, total weight lifted, and speed than females [72]. This fact could be related to the higher capacity of males to delay the onset of muscle pain and reduce muscle damage during exercise compared to females [88]. From a hormonal perspective, females may obtain minor ergogenic effects fromcaffeine intake due to the interaction of caffeine and females’ sex hormones [11]. Likewise, the greater effect of caffeine in individuals with a larger muscle mass could explain the above finding [15], as females have less muscle mass than males [65]. Regarding this difference in body mass and body composition between sexes, it was suggested that females should receive more caffeine than males [111]. However, Arazi et al. [65] showed that both 2 mg·kg^−1^ and 5 mg·kg^−1^of caffeine supplemented 1h before exercising were not able to improve the performance of female karate athletes. Thus, the above suggestion needs future certification using direct male-female comparison. Additionally, it has been reported that the rate of caffeine metabolism varies widely among individuals due to variability in the hepatic CYP1A2 and ADORA2A enzymes, which gives invariable responses to caffeine [22,112]. In this context, it has been hypothesized that, even though they have the same genotypes, females do not metabolize caffeine as quickly as males, and benefit from a longtime between caffeine administration and subsequent tasks [113]. Consequently, it appears that the timing of 1 h prior to testing was insufficient for female athletes to improve their performance as indicated in two trials [55,65]. However, these findings cannot neglect the ergogenic potential of caffeine on female athletes [29], as the effectiveness of caffeine on muscular strength was suggested to be similar for both sexes [31]. It should be noted that among the included studies there was only minor participation of females. This fact could be related to the differences in caffeine metabolism across the follicular and luteal phases of the menstrual cycle, which may increase the complexity of the study design [21]. In just one report comparing taekwondo performance based on sex response to 3 mg·kg^−1^ of caffeine, Ouergui et al. [68] showed no significant interaction between sex and condition, even though males performed better than females. Based on the inconsistent results, more individual studies on this topic are needed before drawing firm conclusions.

#### 4.5.2. Effect of Habitual Intake

Even though habituation is frequently acknowledged as a factor affecting the acute response to caffeine administration [20], research on this issue has produced mixed results [114]. Habitual caffeine intake was reported to reduce the ergogenic effect of acute caffeine ingestion on exercise performance [115]. In this regard, regular caffeine use has been linked to an increase in the number of adenosine receptors in the brain’s vascular and neural tissues [116]. Based on the fact that the recruited athletes in the included studies were regular caffeine consumers, daily consumption may have created a tolerance to the substance, minimizing its performance benefits [20]. An investigation in male judo athletes [50] might corroborate this explanation. The ingestion of three doses of caffeine (i.e., 3, 6 and 9 mg·kg^−1^) by both consumers and non-consumers, resulted in an increase inthe number of attacks in threejudo matches in consumers only following the ingestion of a high dose (9 mg·kg^−1^) of caffeine. However, for non-consumers, the number of attacks improved with moderate (6 mg·kg^−1^), as well as with high (9 mg·kg^−1^) doses. To eliminate caffeine tolerance, it has been proposed that high habitual caffeine users require a pre-exercise caffeine dose above their habitual intake [114]. However, it seems that habitual caffeine consumption effects on its ergogenic potential remain under debate since recent studies [117,118,119] showed the stimulant-like effect of caffeine persists after continuous caffeine consumption. In addition, a previous meta-analysis showed habitual caffeine intake does not negate the benefits of acute caffeine ingestion on resistance exercise [120]. Furthermore, based on the fact that “responders” to caffeine supplementation were not homogenous in terms of daily caffeine consumption, Hudson et al. [121] suggested that studies should compare the characteristics of those who experience ergogenic benefits from caffeine with those who do not.

#### 4.5.3. Effect of Training Status

Caffeine’s ability to alter high-intensity exercise may differ according to the training status [122]. The findings in this context are controversial. It was suggested that the potential effect of improvement after caffeine ingestion decreased with training experience since that athletes have reached their upper limits of exercise performance and physical conditioning [51,123]. However, it is likely that elite athletes have greater motivation to perform a fatiguing exercise and can provide more consistent performance day-to-day [124]. In this regard, it was reported that trained subjects have greater adenosine A_2a_ receptors than untrained ones [125]. In this way, it might be possible that the high density of adenosine receptors for elite athletes allows greater binding of caffeine to these receptors [122], which increases the level of performance enhancement following caffeine ingestion. Additionally, Burke [124] reported that the research sample may have an impact on the findings and that studies using just elite athletes would be most relevant. This is supported by the fact that more familiarity with training would lead to a lower degree of fluctuation, allowing modest changes in performance to be recorded with significance [124]. Thus, there are inconsistent findings concerning the training level effect. From the included studies, no one has compared the response to caffeine administration based on training level, which increases the need for future studieson this topic.

#### 4.5.4. Effect of Age

It was indicated that age may modulate caffeine effects [12,126]. The elderly in particular, are more sensitive to the objective effects (e.g., physical performance) of caffeine while expressing less subjective effects (e.g., RPE) than the young [126]. These findings can be supported by the results from adolescent athletes [45,65], where the subjective perception of effort decreased after both specific (by 14.63% after SJFT) and non-specific tests (leg press) when moderate doses (4–5 mg·kg^−1^) of caffeine were consumed. However, it seems that adolescent athletes may also receive objective benefits from caffeine supplementation as low and moderate doses improved anaerobic performance [45,68]. In the present meta-analysis, age could explain performance variation during the SJFT. Specifically, it was reported that senior athletes demonstrated a higher total number of throws and HR immediately after the SJFT with a lower SJFT index compared to junior athletes [127]. However, in the context of caffeine supplementation, these findings need to be confirmed in future research, as Astley et al. [45] found that teenage athletes who took 4 mg·kg^−1^ of caffeine performed better on the SJFT. This might mean that the response of different ages to caffeine supplementation is depending on the caffeine dosage. Therefore, more research on the influence of varied caffeine dosages on the age’s responses is suggested.

#### 4.5.5. Effects of Genetic Background

In recent decades, the effect of polymorphism on caffeine ergogenic potential has been well studied [22,128,129]. In fact, as determinants of caffeine metabolism, CYP1A2 and ADORA2A were considered as two potential candidates [122]. The contradictory results observed among studies even when the same supplementation protocols were used with similar subjects’ habitual consumption (moderate) could reinforce the notion that responses to caffeine may be triggered by other factors, such as genetics, rather than habitual caffeine intake. However, there was a paucity of sample genetic profile characterization among the 26 included studies. Regarding results from previous studies, there was a large debate concerning the role of CYP1A2 and ADORA2A genes in determining the effects of caffeine on performance [11]. The effects of genotype on performance maybe especially noticeable during longer-duration training or competition, or when fatigue builds up (aerobic or muscular endurance) [22]. Fromthis perspective, the previous meta-analysis suggested that the effects of genetics on the ergogenic effectsof caffeine should be controlled [22]. This was supported in another study [128], which confirmed that the characterization of the athlete’s genetic profile could potentially help in individualizing the caffeine dose accurately to optimize its effect on physical performance. Furthermore, as slower metabolizers are likely to have a prolonged caffeine half-life compared to faster metabolizers [22], identifying an athlete’s genetic profile may be crucial in selecting the best supplement’s timing. However, in a more recent meta-analysis, Grgic reported that polymorphisms within CYP1A2 and ADORA2A do not seem to modulate caffeine’s ergogenic effects on resistance exercise [120] and that there is no high applicability of determining the genetic characteristic of participants in the field of athletic performance. Moreover, because it is unclear which genetic differences affect caffeine’s ergogenic effects, it was suggested that any classification based on genotype is likely to be incomplete [122]. To find more consistent results, further studies on the effect of genetic backgrounds on athletic performance are required.

#### 4.5.6. Effect of Caffeine Dose

The dosage has been reported to explain 16% of the between-study variance [21]. In combat sports, the variance in caffeine dose could also explain the discrepancy and the heterogeneity across studies [8]. For instance, Astley et al. [45] showed that 4 mg·kg^−1^ of caffeine consumed 1 h before performing the SJFT was sufficient to reduce the SJFT index by 22.29%. However, lower doses (i.e., 3 mg·kg^−1^) with similar timing resulted in a lower impact (~5%) [57], whereas a higher dose (5–6 mg·kg^−1^) did not affect the SJFT index [47]. These discrepancies are most likely because the ‘ideal’ caffeine intake is extremely individualized [32]. Moreover, the divergent results related to the effects of varied caffeine doses across different combat sports modalities might be related to the mechanisms that act differently according to caffeine doses. In fact, caffeine low doses (i.e., ≤3 mg·kg^−1^) act mainly on the central nervous system [14,129], maybe by increasing the prefrontal cortex activity and enhancing executive function [130,131]. Such doses of caffeine were suggested to be more effective in long-duration exercises, which are more likely to cause central nervous system fatigue [17]. However, increased doses (i.e., 5–9 mg·kg^−1^) of caffeine may induce peripheral effects [129], failing to affect brain activation [130]. This may be true since the effects of caffeine on cognition and brain activation were greater with low doses of caffeine than with moderate and high doses [131]. Because striking combat sports have shorter high-intensity efforts interspersed by longer pause periods (compared with grappling sports) [1], it is likely that low doses of caffeine (i.e., 2–3 mg·kg^−1^) administrated at one time [49] or repeatedly [54] are sufficient only in the grappling contests (i.e., 6/7 studies in grappling vs. 1/2 in striking that reported improved performances), whereas moderate doses (i.e., 4–6 mg·kg^−1^) are more effective in striking contests (i.e., 5/7 studies in striking vs. 4/12 studies in grappling improved performances).

In the context of caffeine dose, it has been suggested that greater doses of caffeine might be warranted for a performance-enhancing effect when exercising with higher loads [105]. From our data, this was relatively true for some physiological variables (e.g., HR, [La]). However, increasing the caffeine dose should be conducted based on the individual’s tolerance to the substance and the type of physical exercise [12]. Besides, using low doses of caffeine seems to be effective and safe in the context of simulated combat [49].

#### 4.5.7. Effect of Supplementation Timing and Form

As indicated in the previous summary [11], the most common timing of caffeine supplementation is 60 min before exercise. This choice of this supplementation timing was explained by the peak plasma levels of caffeine at this timing [16]. This fact could explain, in part, the lack of significant results in previous studies [44,63] using less than 1h post-supplementation. This could be evident, as all studies used a single administrated dose. However, Negaresh et al. [54] reported that during a simulated wrestling tournament, administering caffeine to individuals based on their physical performance decrement (i.e., selective caffeine supplementation) induced the highest improvements in performance, particularly, during the last competition. However, the optimal timing of caffeine supplementation likely depends on the source of caffeine [11]. Capsules are the most frequent ways to obtain caffeine as a supplement [105]. In the present meta-analysis, an alternative method of caffeine administration via chewing gum has been proposed [51]. Such a form of caffeine administration has been suggested to have an earlier onset of pharmacological effects [11]. Caffeine delivered in the form of chewing gum may hasten the rate of caffeine absorption into the blood stream via the highly vascular buccal cavity, resulting in an early increase in blood plasma caffeine concentration, usually between 5 and 15 min after administration [132]. However, Filip-Stachnik et al. [51] reported that both 2.7 mg·kg^−1^ and 5.4 mg·kg^−1^ caffeinated chewing gum supplemented 15 min before performing two SJFT did not improve the total number of throws and the test index. Further studies comparing the different forms of caffeine supplementation, such as caffeinated chewing gum, mouth rinses, aerosols, inspired powders, energy bars, energy gels and chews could be of interest.

#### 4.5.8. Effects of Time of Day

The morning-evening performance gap, as well as the potential moderating influence of caffeine use, is an important methodological aspect for researchers to report in future investigations [122]. The effect of time of day on caffeine erogeneity has been investigated with greater effects observed in the morning [133,134]. Because of its stimulant properties, caffeine maybe used to counteract the morning performance fall [135]. These benefits were presented asanincrease invigor, a reduction of the simple reaction time and an improvement of the peak and mean power values during the Wingate test [136], improvement in cognitive functions [135], and counteraction of the effect of sleep deprivation [137,138,139]. Of the 26 included studies, only eight [45,48,49,51,60,61,64,66] identified the timeofday at which the testing exercise was performed. Therefore, future investigations should pay attention to the effect of time of day, as this could be one of the factors that modulate caffeine impact. Captivatingly, as Pickering and Grgic [122] note, it would be interesting to see if an individual’s chronotype alters the ergogenic benefits of caffeine throughout different times in the day.

### 4.6. Adverse Effects

Excessive doses of caffeine consumption are known to induce a high incidence of side effects [11,124], such as tachycardia, anxiety, gastrointestinal discomfort, tremors, hypertension, and insomnia [119]. Among the included studies, Lopes-Silva et al. [66] reported caffeine produced an increase in the tachycardia sensation in 20% of athletes and anxiety/nervousness in 10% of athletes. In addition, Felippe et al. [58] investigated the possible gastrointestinal discomfort caused by a combined supplementation of sodium bicarbonate (NaHCO_3_) and caffeine. In this study, the authors showed that only one athlete reported flatulence following supplementation [58]. However, this gastrointestinal discomfort was attributed to the ingestion of NaHCO_3_, but not to caffeine. To avoid the negative effects of NaHCO_3_ when it was administrated alone or with caffeine, it was suggested that ingesting divided NaHCO_3_ doses is the best strategy [58,63]. However, among freestyle wrestlers, the supplementation with high doses of caffeine alone increased urine volume and the dehydration index when compared to all other conditions [54]. The same study showed the scores of gastrointestinal complaints and gastrointestinal discomfort were higher with the high dose of caffeine and the repeated doses of caffeine, in comparison with the other conditions [54]. Moreover, in BJJ athletes, it was reported that caffeine administration increased the rate of fatigue sensation even when low doses (i.e., 3 mg·kg^−1^) were administered [48].

### 4.7. Methodological Recommendations

As assessed using the Pedro scale, the included studies are classified as being of good or excellent methodological quality. However, in these studies, there was minor participation of females. Possibly, the assessment of the effect of caffeine on female athletes is considered complex as both oral contraceptives and the different phases of the menstrual cycle are designated to be factors that may affect caffeine metabolism [140]. Therefore, the menstrual cycle phases are one methodological element to keep in mind when conducting research on female athletes [140]. However, among the six studies including female athletes [53,55,57,58,63,66], only one [68] has indicated that menstrual cycle phases were considered as an exclusion/inclusion criterion for female athletes. Moreover, despite sex beingan effective moderator that can influence the outcomes, four studies [51,59,61,81] did not indicate the sex of their athletes. Thus, future studies should identify the sex of participants explicitly.

Caffeine habituation is difficult to be quantified accurately [141]. In the 26 included studies, it seems that the dose of caffeine consumed per day was given as an approximation. However, measuring objective markers, such as urine caffeine production, plasma caffeine, or caffeine metabolite levels, could be a solution [142]. Otherwise, future studies should ensure that a validated questionnaire for assessing habitual caffeine intake is used (e.g., the questionnaire proposed by Bühler et al. [143]). Additionally, there is no clear classification concerning habitual caffeine intake that can help to categorize athletes into light, moderate and high consumers. Only in two studies [50,68], there was a clear classification. In this context, Durkalec-Michalski et al. [50] considered athletes who consumed less than 160 mg·kg^−1^ as light consumers, whereas, those who consumed more were classified as high consumers. However, Filip et al. [144] proposed a recent classification according to individual’s habitual caffeine consumption with six categories [i.e., Naïve consumer (<25 mg/day); Low consumer (25 mg/day to 0.99 mg/kg/day); Mild consumer (1.00–2.99 mg/kg/day); Moderate consumer (3.00–5.99 mg/kg/day); High consumer (6.00–8.99 mg/kg/day); Very high consumer (>9.00 mg/kg/day)]. Thus, future researchers should consider this categorization to minimize possible methodological bias.

Regarding power analysis, it is interesting to highlight the fact that sample sizes recruited in the majority of the included studies were less than 20 participants, which often limited the statistical power to detect significant effects [145]. Therefore, it is recommended to recruit larger samples in future investigations.

Finally, in some circumstances, giving a placebo with the implication that it is caffeine has been demonstrated to improve performance and reduce RPE [105]. In this regard, Grgic [30] attributed the discrepancy between studies to the effect of an error in the measurement rather than the effects of the condition; the success of blinding could determine the result. Among the 26 included studies, only three have tested the success of blinding. Therefore, future research should consider the effectiveness of blinding.

### 4.8. Strengths, Limitations and Perspectives

This is the first systematic review with meta-analysis that addressed the effects of caffeine supplementation on combat sports physical performance, technical-tactical skills, perceived exertion and physiological response. The strengths of the present investigation include a comprehensive exposure of the available literature and a careful appraisal of its value. Moreover, the search was conducted without any language or year restriction. In addition, the study was conducted according to the updated PRISMA guidelines. Furthermore, the recommendations issued from this study can help to draw strong investigations into the topic of caffeine supplementation and athletic performance, particularly, in the context of combat sports. However, the present systematic review presents some limitations related to the inclusion criteria. Indeed, investigations in which caffeine was co-ingested with other substances (i.e., sodium bicarbonate) were selected, which can lead to other effects. Moreover, meta-analytical calculations were not possible for the different outcomes, due to the limited number of studies, and diverse methodology. Furthermore, meta-regression and subgroup analysis were not performed, respecting the Cochrane handbook recommendations [39]. Further studies, such as those using a mixed-sex sample and undertaking between-group comparisons, are warranted. In addition, recruiting samples with different competitive levels and ages could help to generalize the effect of caffeine administration on combat sports’ performance atdifferent expertise levels or ages. Moreover, in the included studies, only three compared the effects of low and moderate doses of caffeine. Thus, future protocols should be designed to compare the effects of caffeine depending on the dose and form, particularly in striking combat sports. Furthermore, because combat sports are contact sports where tactical behaviors are needed, further investigations are necessary to determine the effects of caffeine supplementation in real competition outcomes.

The improvement in performance following caffeine supplementation, without affecting the metabolism process in some studies [53,63], could be informative of other physiological responses than glycogen use. For example, the improvement in physical performance was associated with a 12% increase in testosterone concentration [146]. This hormone is responsible for the increase in risk-taking behaviors and aggressiveness [146], which are considered necessary responses to improve performances in both striking [67] and grappling [49] contests. However, information about testosterone variation in response to caffeine supplementation remains speculative, and future works are warranted.

## 5. Conclusions

The effectiveness of caffeine supplementation may be masked under some conditions, but the possibility of inter-individual variability does not diminish the importance of applying caffeine supplementation in combat sports, particularly when the objective is to enhance specific performance during training and in competition. Supplementation with caffeine served to acutely enhance many aspects of exercise, including isometric strength, anaerobic power, and reaction time, as well as improved anaerobic metabolism of combat sports athletes.

## Figures and Tables

**Table 1:** Terms combinations and search results on each database.

Database	Terms Combination	Results
Pubmed	(“Caffeine”[Mesh]) OR “Coffee” [Mesh] AND “Martial Arts”[Mesh]	19
Web of Science	Caffeine AND Combat Sports	35
Scopus	Caffeine OR Coffee AND Combat Sports OR Martial Arts OR Judo OR Taekwondo OR Wrestling OR Boxing OR Jiu-Jitsu	19
Cochrane Library	Caffeine AND Martial Arts	19

**Table 2:** Summary of the studies examining the impacts of acute caffeine supplementation on combat sports performance [44,45,46,47,48,49,50,51,52,53,54,55,56,57,58,59,60,61,62,63,64,65,66,67,68,69].

Study	Design	Sample Size	Timing	Doses	Form	Measures	Results	Pedro Score
Aedma et al. [44]	RDBCCrSD	14 BJJ practitioners	30 min	5 mg·kg^−1^	Capsules	4 ×arm ergometer test (6 × 15 s + 40 s rest) [La], HR, RPF, RPE	Significant time effect but no treatment ≠ in PP, MP, RPE and RPF.	10
Arazi et al. [65]	RDBCCrSD	10 karatekas	60 min	2 mg·kg^−1^ 5 mg·kg^−1^	Capsules	leg press Vertical jump (sergeant) RAST (6 × 35 m) RPE, pain perception	5 mg·kg^−1^ of caffeine ↓ RPE and pain perception values during muscular endurance test. No ≠ in leg press, vertical jump and RAST performance.	10
Astley et al. [45]	RDBSD	18 judo athletes	60 min	4 mg·kg^−1^	Cgapsules	SJFT HR RPE	Number of throws ↑ by 31.22% SJFT index ↓ by 22.29% RPE ↓ by 14.63% No changes in HR	10
Cortez et al. [61]	RDBSD	13 taekwondo athletes	60 min	5 mg·kg^−1^	Capsules	3 × dollyochagi circular kick pre- and post-3 × 60 s CMJ	Reaction time ↑ by 29% in pretest and by 25% in posttest	10
Coswig et al. [62]	RDBCCrSD	10 boxers	30 min	6 mg·kg^−1^	Capsules	-Simulated boxing matches HR RPE	Duration of interaction blocks↑by 47.73% Effort: Pause ratio was 4.4 s:8.7 s (approx. 1:2) for placebo and 6.5 s:9.4 s (1:1.44) for caffeine No ≠ in HR and RPE between conditions	10
Athayde et al. [46]	RDBCrSD	14 judo athletes	60 min	5 mg·kg^−1^	Capsules	CMJ Handgrip strength Judogi grip strength test 3 × 5-min judo matches separated by 15 min of passive rest [La]	Peak [La] ↑ in the 5th minute after match 3 by 22.14% No effects on CMJ, handgrip strength and the number of attacks.	10
Athayde et al. [56]	RDBCrSD	12 judo athletes	60 min	5 mg·kg^−1^	Capsules	3 × 5-min judo matches separated by 15 min of passive rest RPE and RPR	No effects on match-derived technical variables, RPE and RPR.	10
Diaz-Lara, Del Coso, García et al. [48]	RDBCrSD	14 elite BJJ athletes	60 min	3 mg·kg^−1^	Capsules	Handgrip strength CMJ Maximal static lift Bench-press	Hand grip strength ↑ in dominant hand by 4.4%, and non-dominant hand by 4.9%. CMJ height ↑ by 2.7%. Bench press ↑ by 3%. Max number of bench press reps at maximal strength ↑ by 14.7%	10
Diaz-Lara, Del Coso, Portillo et al. [49]	RDBSD	14 elite BJJ athletes	60 min	3 mg·kg^−1^	Capsules	Handgrip strength Maximum static lift CMJ 2 simulated BJJ combats (with 20 min rest) [La] RPE	The duration of high-intensity offensive actions ↑ in combat 1 by 58.3% and by 42% in combat 2. The number of successful offensive actions↑ in combat 2 by 28.57%. The number of blocks ↑ by 66.7% in combat 1 and by 28.57% in combat 2. Maximal static lift test ↑ by in pre-fight 2 by 15.8% and after fight 2 by 17.8%. Jump height and Handgrip Strength ↑ as in Diaz-Lara et al. [48]. [La] ↑ by 17.33% (8.8 ± 2.9 vs. 7.5 ± 2.7 mmol/L) in the prefight 2, and just after fight 2 by 11.76% (15.2 ± 3.3 vs. 13.6 ± 4.0 mmol/L). PRE ↑ by 4.84%.	10
Durkalec-Michalski et al. [50]	RDBCrSD	22 judo athletes	60 min	3 mg·kg^−1^ 6 mg·kg^−1^ 9 mg·kg^−1^	Capsules	3 × 4-min judo matches SJFT HR RPE	The 6 and 9 mg·kg^−1^ doses ↑the total number of attacks in SJFTs compared to 3 mg·kg^−1^, PLA or baseline. RPE remains unchanged. 9 mg·kg^−1^CAF ↑ HR_RA_ and HR_1minAF_ SJFTs as compared to PLA. Total number of throws in randoris were ↑ with 9 mg·kg^1^ compared to PLA.	10
Merino Fernández et al. [60]	RDBCrSD	16 Spanish national Jiu-Jitsu athletes	60 min	3 mg·kg^−1^	Capsules	3 CMJ with both legs, 3 CMJ with the right leg and 3 CMJ with the left leg	Caffeine ↑bilateral jump height (Δ% = 4.40), flight time (Δ% = 2.20), flight time: contraction time (Δ% = 8.90), concentric impulse (Δ% = 1.80), peak power (Δ% = 2.50). Caffeine ↑left leg flight time (Δ% = 1.91), left leg jump height (Δ% = 3.75) and right leg flight time: contraction time (Δ% = 9.72).	10
Merino Fernández et al. [57]	RDBSD	22 jiu-jitsu athletes	60 min	3 mg·kg^−1^	Capsule	SJFT Simulated combat HR	Number of throws during the SJFT ↑ by 5%. SJFT index ↓ by 5.8%. HR during SJFT ↑ by 4%. No ≠ for offensive and defensive technical actions.	10
Krawczyk et al. [59]	RDBCrSD	10 judo athletes	60 min	3 mg·kg^−1^ 6 mg·kg^−1^	Capsules	bench press with 50% of 1RM bench pull with 50% of 1RM CMJ Handgrip strength Dynamic and isometric versions of the Judogi Grip Strength Test	Both 3 and 6 mg·kg^−1^ of caffeine: ↑ Peak bar velocity in the bench press by 5.2%. ↑Mean bar velocity in the bench pull by ~ 9% and 12%. ↑Number of repetitions in the Judogi Grip Strength Test by 15%. No ≠ in the CMJ and handgrip strength.	10
Pereira et al. [55]	RDBCrSD	13 judo athletes	60 min	6 mg·kg^−1^	Capsules	SJFT HR	No ≠ in number of throws, SJFT index and HR.	10
San Juan et al. [64]	RDBCCrSD	8 boxer athletes	60 min	6 mg·kg^−1^	Capsules	Handgrip strength CMJ 30-s Wingate test [La]	Caffeine ↑ peak power by 6.27%, ↓ The time to reach peak power by −9.91% in the Wingate test, ↑ jump height by 5.1%. Enhance neuromuscular efficiency at peak power in the vastus lateralis and gluteus maximus.	10
Santos et al. [67]	RDBCrSD	10 taekwondo athletes	60 min	5 mg·kg^−1^	Capsules	2 × (5 × bandaltchagui kick) + 2 simulated taekwondo matches). [La]; HR; RPE	caffeine ↑ the reaction time by 11.9% prior to the first combat. ↑ number of attacks in combat 2 by 37.39%. ↑ referee time-outs in combat 2 by 60%. ↑ [La] after round 2 by 31.65% and by 35.48% in the first combat. RPE and HR were unaffected.	10
Lopes-Silva et al. [53]	RDBCrSD	10 judo and jiu-jitsu athletes	60 min	5 mg·kg^−1^	Capsules	4 set of judogi dynamic strength-endurance test Handgrip strength [La]; HR; RPE	Caffeine ↑ the number of repetitions by 7% and ↑ maximal isometric handgrip strength by 5%. [La]; HR and RPE values were unaffected.	10
Lopes-Silva et al. [52]	RDBCCrSD	6 judo athletes	60 min	6 mg·kg^−1^	Capsules	3 × SJFT [La] RPE	No significant ≠ in the number of throws. [La] was systemically higher and RPE ↓ by 14.6% with caffeine.	10
Lopes-Silva et al. [66]	RDBCrSD	10 taekwondo athletes	60 min	5 mg·kg^−1^	Capsules	3 rounds of 2 min [La] HR RPE	No ≠ in any of the variables examined.	10
Carmo et al. [47]	RDBCrSD	8 judo athletes	60 min and 120 min (post-traning)	5 mg·kg^−1^	Capsules	SJFT CMJ [La], HR RPE, Pain perception	At 120 min: [La] ↑ by 63.63%. No ≠ in number of throws and SJFT index No ≠ for HR, CMJ, perceived pain and RPE.	9
Filip-Stachnik et al. [51]	RDBCrSD	9 judo athletes	15 min	5.4 mg·kg^−1^ 2.7 mg·kg^−1^	Caffeinated chewing gums	2 × SJFT separated by 4 min of combat RPE HR [La]	No ≠ in number of throws, SJFT index, [La], HR and RPE.	10
Felippe et al. [58]	RDBCSD	10 judo athletes	60 min	0.3 g·kg^−1^ of NaHCO_3_ 6 mg·kg^−1^ of caffeine NaHCO_3_+ caffeine	Capsules	3 × SJFT RPE [La]	Caffeine+ NaHCO_3_: ↑ Number of throws in SJFT1 by 5.17%, in SJFT3 by 8.44% and total number of throws by 5.66%. Caffeine ↑ [La] in all experimental conditions compared with placebo. No significant ≠ in RPE	10
de Azevedo et al. [69]	RDBCCrSD	11 MMA athletes	60 min	5 mg·kg^−1^	Capsules	3 sets of repeated punching (15 s + 45 s rest) RPE	No ≠ in the number of throws and RPE.	10
Rezaei et al. [63]	RDBCrSD	8 karatekas	50 min	5 mg·kg^−1^ NaHCO_3_	Capsules	Karate aerobic specific test (KAST) [La]; RPE; HR	Caffeine ↓ time to exhaustion by 5.97%. HR, RPE and [La] were unaffected.	10
Negaresh et al. [54]	RDBSD	11 freestyle wrestlers	45 min before 1st match ~30 min before the following matches	Selective dose 10 mg·kg^−1^ 4 mg·kg^−1^ Repeated-dose (5 × 2 mg·kg^−1^)	Capsules	Pittsburgh Wrestling Performance Test (PWPT) 5 wrestling matches (2 × 3-min wrestling rounds) HR [La] RPF	In comparison to the Placebo: (10 mg·kg^−1^ of caffeine ↓ PWPT time only before the 1st match. The repeated dose and the selective dose reduced PWPT time in the 3rd and 4th matches. RPF was less before the 4th match with the repeated and selective dose [La] was higher with the selective dose after the 4thand 5th matches.	10
Ouergui et al. [68]	RDBCCrSD	20 taekwondo athletes	60 min	3 mg·kg^−1^	Capsules	TSAT FSKT-10s FSKT-mult RPE	↓ TSAT time by ~3.3% ↑ FSKT-10s by 4% No significant ≠ in FSKT-mult and RPE	10

↓: Decrease; ↑: Increase; ≠: No difference; SJFT: Special Judo Fitness Test; HR: Heart Rate; RPE: Rating of Perceived Exertion; [La]: Lactate concentration; RPF: Ratings of perceived fatigue, BJJ: Brazilian jiu-jitsu; PP: Peak Power; MP: Mean Power; TSAT: Taekwondo Specific Agility Test; FSKT-10s: 10 s Frequency Speed of Kick Test; FSKT-mult: Multiple Frequency Speed of Kick Test; RAST: Repeated anaerobic Sprint Test; T: Time; CMJ: Countermovement jump; NaHCO_3_: Sodium Bicarbonate; 1RM: One-Repetition Maximum; RDBCCrSD: Randomized Double Blind counterbalanced Crossover Study Design; RDBCSD: Randomized Double Blind counterbalanced Study design; RDBCrSD: Randomized Double Blind Crossover Study Design; RDBSD: Randomized Double Blind Study Design.

**Table 3:** Characteristics of the population investigated in different studies [44,45,46,47,48,49,50,51,52,53,54,55,56,57,58,59,60,61,62,63,64,65,66,67,68,69].

Study	Sex	Habitual Caffeine Intake	Age (Years)	Experience (Years)
Aedma et al. [44]	NR	10 intake < 70 and 4 were intake 80–200 mg/day.	25.3 ± 4.9	≥4
Arazi et al. [65]	F	˂60 mg/day, and 1 cup of coffee	16.8 ± 1.2	≥3
Astley et al. [45]	M	NR	16.1 ± 1.4	NR
Athayde et al. [46]	M	Moderate caffeine consumers	22.5 ± 7.1	12.9 ± 6.4
Athayde et al. [56]	M	Moderate caffeine consumers	23.1 ± 4.2	12.5 ± 3.9
Carmo et al. [47]	M	NR	21.6 ± 2.0	≥7
Cortez et al. [61]	NR	NR	22.8 ± 4.7	3.7 ± 1.8
Coswig et al. [62]	M	NR	25.9 ± 5.2	5 ± 1.5
de Azevedo et al. [69]	M	<250 mg/day	27.6 ± 4.3	NR
Diaz-Lara, Del Coso, García et al. [48]	M	<60 mg/day	29.2 ± 3.3	≥5
Diaz-Lara, Del Coso, Portillo et al. [49]	M	Light caffeine consumers	29.2 ± 3.3	≥5
Durkalec-Michalski et al. [50]	M	10 consumers 12 non-consumers	21.7 ±3.7	11.0 ± 4.5
Felippe et al. [58]	M	<2 cups of coffee/day	23 ± 5	15 ± 5
Filip-Stachnik et al. [51]	M	3.1 ± 1.3 mg/day	23.7 ± 4.4	15.6 ± 4.0
Lopes-Silva et al. [53]	NR	NR	25.2 ± 5.3	≥10
Lopes-Silva et al. [52]	M	NR	25.3 ± 5.7	14.4 ± 8.9
Lopes-Silva et al. [66]	M	2 consumed > 6 cups/day 8 consumed < 2 cups/day	21 ± 4	≥9
Merino Fernández et al. [60]	M F	<0.99 mg/day	21.50 ± 4.75 20.63 ± 3.20	11.88 ± 3.94 15.38 ± 2.92
Merino Fernández et al. [57]	M/F	NR	22 ± 4	15 ± 7
Krawczyk et al. [59]	M/F	2.6 mg/kg/day	24.1 ± 4.7	15.1 ± 5.2
Negaresh et al. [54]	M	<3 cups of coffee/day	24 ± 3	≥10
Pereira et al. [55]	F	NR	17.6 ± 1.6	≥2
Rezaei et al. [63]	NR	≤125 mg/day	20.5 ± 2.4	>5
San Juan et al. [64]	M	NR	22.0 ± 1.78	NR
Santos et al. [67]	M	NR	24.9 ± 7.3	≥7
Ouergui et al. [68]	M/F	<3 cups of coffee/day	17.5 ± 0.7	≥6

M: Male; F: Female; NR: Not Reported.

## Data Availability

Data that support the findings of this study are available from the corresponding author upon reasonable request.

## Supplementary Materials

The following supporting information can be downloaded at: https://www.mdpi.com/article/10.3390/nu14142996/s1, Table S1: List of excluded studies in each level; Table S2: Results of the Begg and Mazumdar’s Rank Correlation Test and Egger’s Linear Regression Test; Figure S1: Funnel plot for CMJ showing no evidence of publication bias; Figure S2: Funnel plot for handgrip strength showing no evidence of publication bias; Figure S3: Funnel plot for SJFT number of throw showing no evidence of publication bias; Figure S4: Funnel plot for SJFT index showing an evidence of publication bias; Figure S5: Funnel plot for judogi strength endurance test showing no evidence of publication bias; Figure S6: Funnel plot for [La] post-anaerobic exercise showing an evidence of publication bias; Figure S7: Funnel plot for [La] post-combat showing an evidence of publication bias; Figure S8: Funnel plot for HR final showing no evidence of publication bias; Figure S9: Funnel plot for HR 1min showing no evidence of publication bias; Figure S10: Funnel plot for HR post-combat showing no evidence of publication bias; Figure S11: Funnel plot for RPE post-anaerobic test showing no evidence of publication bias; Figure S12: Funnel plot for RPE post-combat showing no evidence of publication bias; Figure S13: Funnel plot for offensives actions showing no evidence of publication bias; Figure S14: Funnel plot for offensives actions duration showing no evidence of publication bias; Figure S15: Forest plot of the leave-one-out sensitivity analysis for CMJ; Figure S16: Forest plot of the cumulative meta-analysis for CMJ; Figure S17: Forest plot of the leave-one-out sensitivity analysis for handgrip-strenght; Figure S18: Forest plot of the cumulative meta-analysis for handgrip-strenght; Figure S19: Forest plot of the leave-one-out sensitivity analysis for toatal number of throws during the SJFT; Figure S20: Forest plot of the cumulative meta-analysis for toatal number of throws during the SJFT; Figure S21: Forest plot of the leave-one-out sensitivity analysis for SJFT index; Figure S22: Forest plot of the cumulative meta-analysis for SJFT index; Figure S23: Forest plot of the leave-one-out sensitivity analysis for the judogi strength endurance test; Figure S24: Forest plot of the cumulative meta-analysis for the judogi strength endurance test; Figure S25: Forest plot of the leave-one-out sensitivity analysis for [La] post-anaerobic exercise; Figure S26: Forest plot of the cumulative meta-analysis for [La] post-anaerobic exercise; Figure S27: Forest plot of the leave-one-out sensitivity analysis for [La] post-combat; Figure S28: Forest plot of the cumulative meta-analysis for [La] post-combat; Figure S29: Forest plot of the leave-one-out sensitivity analysis for HR final; Figure S30: Forest plot of the cumulative meta-analysis for HR final; Figure S31: Forest plot of the leave-one-out sensitivity analysis for HR 1 min; Figure S32: Forest plot of the cumulative meta-analysis for HR 1 min; Figure S33. Forest plot of the leave-one-out sensitivity analysis for HR at the end-of-fight; Figure S34: Forest plot of the cumulative meta-analysis for HR at the end-of-fight; Figure S35: Forest plot of the leave-one-out sensitivity analysis for RPE post-anaerobic exercise; Figure S36: Forest plot of the cumulative meta-analysis for RPE post-anaerobic exercise; Figure S37: Forest plot of the leave-one-out sensitivity analysis for RPE post-combat; Figure S38: Forest plot of the cumulative meta-analysis for RPE post-combat; Figure S39: Forest plot of the leave-one-out sensitivity analysis for the number of offensives actions; Figure S40: Forest plot of the cumulative meta-analysis for the number of offensives actions; Figure S41: Forest plot of the leave-one-out sensitivity analysis for the duration of offensives actions; Figure S42: Forest plot of the cumulative meta-analysis for the duration of offensives actions; PRISMA 2020 Main Checklist; and PRISMA Abstract Checklist [38].

## References

[1] Franchini E., Cormack S., Takito M.Y. (2019). Effects of High-Intensity Interval Training on Olympic Combat Sports Athletes’ Performance and Physiological Adaptation: A Systematic Review. J. Strength Cond. Res..

[2] Vasconcelos B.B., Protzen G.V., Galliano L.M., Kirk C., Del Vecchio F.B. (2020). Effects of High-Intensity Interval Training in Combat Sports: A Systematic Review with Meta-Analysis. J. Strength Cond. Res..

[3] Bridge C.A., Ferreira da Silva Santos J., Chaabène H., Pieter W., Franchini E. (2014). Physical and physiological profiles of taekwondo athletes. Sports Med..

[4] Franchini E., Del Vecchio F.B., Matsushigue K.A., Artioli G.G. (2011). Physiological profiles of elite judo athletes. Sports Med..

[5] Krabben K., Orth D., van der Kamp J. (2019). Combat as an interpersonal synergy: An ecological dynamics approach to combat sports. Sports Med..

[6] Simoncini L., Lago-Rodriguez A., Lopez-Samanes A., Perez-Lopez A., Dominguez R. (2021). Effects of nutritional supplements on judo-related performance: A review. J. Hum. Kinet..

[7] Begum G., Cunliffe A., Leveritt M. (2005). Physiological role of carnosine in contracting muscle. Int. J. Sport Nutr. Exerc. Metab..

[8] Lopez-Gonzalez L.M., Sanchez-Oliver A.J., Mata F., Jodra P., Antonio J., Dominguez R. (2018). Acute caffeine supplementation in combat sports: A systematic review. J. Int. Soc. Sports Nutr..

[9] Maughan R. (2018). Dietary supplements and the high-performance athlete. Int. J. Sport Nutr. Exerc. Metab..

[10] Del Coso J., Munoz G., Munoz-Guerra J. (2011). Prevalence of caffeine use in elite athletes following its removal from the World Anti-Doping Agency list of banned substances. Appl. Physiol. Nutr. Metab..

[11] Guest N.S., VanDusseldorp T.A., Nelson M.T., Grgic J., Schoenfeld B.J., Jenkins N.D.M., Arent S.M., Antonio J., Stout J.R., Trexler E.T. (2021). International society of sports nutrition position stand: Caffeine and exercise performance. J. Int. Soc. Sports Nutr..

[12] Martins G.L., Guilherme J., Ferreira L.H.B., de Souza-Junior T.P., Lancha A.H. (2020). Caffeine and exercise performance: Possible directions for definitive findings. Front. Sports Act. Living.

[13] Astorino T.A., Roberson D.W. (2010). Efficacy of acute caffeine ingestion for short-term high-intensity exercise performance: A systematic review. J. Strength Cond. Res..

[14] Wang C., Zhu Y., Dong C., Zhou Z., Zheng X. (2020). Effects of various doses of caffeine ingestion on intermittent exercise performance and cognition. Brain Sci..

[15] Woolf K., Bidwell W.K., Carlson A.G. (2008). The effect of caffeine as an ergogenic aid in anaerobic exercise. Int. J. Sport Nutr. Exerc. Metab..

[16] Graham T.E. (2001). Caffeine and exercise: Metabolism, endurance and performance. Sports Med..

[17] Davis J.K., Green J.M. (2009). Caffeine and anaerobic performance: Ergogenic value and mechanisms of action. Sports Med..

[18] Davis J.M., Zhao Z., Stock H.S., Mehl K.A., Buggy J., Hand G.A. (2003). Central nervous system effects of caffeine and adenosine on fatigue. Am. J. Physiol. Regul. Integr. Comp. Physiol..

[19] Doherty M., Smith P.M. (2005). Effects of caffeine ingestion on rating of perceived exertion during and after exercise: A meta-analysis. Scand. J. Med. Sci. Sports.

[20] Daly J. (2007). Caffeine analogs: Biomedical impact. Cell Mol. Life Sci..

[21] Grgic J., Grgic I., Pickering C., Schoenfeld B.J., Bishop D.J., Pedisic Z. (2020). Wake up and smell the coffee: Caffeine supplementation and exercise performance-an umbrella review of 21 published meta-analyses. Br. J. Sports Med..

[22] Southward K., Rutherfurd-Markwick K., Badenhorst C., Ali A. (2018). The role of genetics in moderating the inter-individual differences in the ergogenicity of caffeine. Nutrients.

[23] Souza D.B., Del Coso J., Casonatto J., Polito M.D. (2017). Acute effects of caffeine-containing energy drinks on physical performance: A systematic review and meta-analysis. Eur. J. Nutr..

[24] Grgic J., Trexler E.T., Lazinica B., Pedisic Z. (2018). Effects of caffeine intake on muscle strength and power: A systematic review and meta-analysis. J. Int. Soc. Sports Nutr..

[25] Warren G.L., Park N.D., Maresca R.D., McKibans K.I., Millard-Stafford M.L. (2010). Effect of caffeine ingestion on muscular strength and endurance: A meta-analysis. Med. Sci. Sports Exerc..

[26] Christensen P.M., Shirai Y., Ritz C., Nordsborg N.B. (2017). Caffeine and bicarbonate for speed. A meta-analysis of legal supplements potential for improving intense endurance exercise performance. Front. Physiol..

[27] Ferreira T.T., da Silva J.V.F., Bueno N.B. (2021). Effects of caffeine supplementation on muscle endurance, maximum strength, and perceived exertion in adults submitted to strength training: A systematic review and meta-analyses. Crit. Rev. Food Sci. Nutr..

[28] Glaister M., Gissane C. (2018). Caffeine and physiological responses to submaximal exercise: A meta-analysis. Int. J. Sports Physiol Perform..

[29] Gomez-Bruton A., Marin-Puyalto J., Muniz-Pardos B., Matute-Llorente A., Del Coso J., Gomez-Cabello A., Vicente-Rodriguez G., Casajus J.A., Lozano-Berges G. (2021). Does acute caffeine supplementation improve physical performance in female team-sport athletes? Evidence from a systematic review and meta-analysis. Nutrients.

[30] Grgic J. (2018). Caffeine ingestion enhances Wingate performance: A meta-analysis. Eur. J. Sport Sci..

[31] Grgic J., Del Coso J. (2021). Ergogenic effects of acute caffeine intake on muscular endurance and muscular strength in women: A meta-analysis. Int. J. Environ. Res. Public Health.

[32] Grgic J., Diaz-Lara F.J., Coso J.D., Duncan M.J., Tallis J., Pickering C., Schoenfeld B.J., Mikulic P. (2020). The effects of caffeine ingestion on measures of rowing performance: A systematic review and meta-analysis. Nutrients.

[33] Grgic J., Mikulic P. (2021). Effects of caffeine on rate of force development: A meta-analysis. Scand. J. Med. Sci. Sports.

[34] Raya-Gonzalez J., Rendo-Urteaga T., Dominguez R., Castillo D., Rodriguez-Fernandez A., Grgic J. (2020). Acute Effects of Caffeine Supplementation on Movement Velocity in Resistance Exercise: A Systematic Review and Meta-analysis. Sports Med..

[35] Salinero J.J., Lara B., Del Coso J. (2019). Effects of acute ingestion of caffeine on team sports performance: A systematic review and meta-analysis. Res. Sports Med..

[36] Grgic J. (2022). Effects of caffeine on isometric handgrip strength: A meta-analysis. Clin. Nutr. ESPEN.

[37] Diaz-Lara J., Grgic J., Detanico D., Botella J., Jiménez S.L., Del Coso J. (2022). Effects of acute caffeine intake on combat sports performance: A systematic review and meta-analysis. Crit. Rev. Food Sci. Nutr..

[38] Page M.J., McKenzie J.E., Bossuyt P.M., Boutron I., Hoffmann T.C., Mulrow C.D., Shamseer L., Tetzlaff J.M., Akl E.A., Brennan S.E. (2021). The PRISMA 2020 statement: An updated guideline for reporting systematic reviews. J. Clin. Epidemiol..

[39] Higgins J.P., Thomas J., Chandler J., Cumpston M., Li T., Page M.J., Welch V.A. (2019). Cochrane Handbook for Systematic Reviews of Interventions.

[40] Trabelsi K., Bragazzi N., Zlitni S., Khacharem A., Boukhris O., El-Abed K., Ammar A., Khanfir S., Shephard R.J., Hakim A. (2020). Observing Ramadan and sleep-wake patterns in athletes: A systematic review, meta-analysis and meta-regression. Br. J. Sports Med..

[41] Trabelsi K., Ammar A., Boukhris O., Glenn J.M., Clark C.C.T., Stannard S.R., Slater G., Zmijewski P., Driss T., Ben Saad H. (2022). Dietary intake and body composition during Ramadan in athletes: A systematic review and meta-analysis with meta-regression. J. Am. Nutr. Assoc..

[42] Rico-González M., Pino-Ortega J., Clemente F., Los Arcos A. (2022). Guidelines for performing systematic reviews in sports science. Biol. Sport.

[43] Buscemi N., Hartling L., Vandermeer B., Tjosvold L., Klassen T.P. (2006). Single data extraction generated more errors than double data extraction in systematic reviews. J. Clin. Epidemiol..

[44] Aedma M., Timpmann S., Ööpik V. (2013). Effect of caffeine on upper-body anaerobic performance in wrestlers in simulated competition-day conditions. Int. J. Sport Nutr. Exerc. Metab..

[45] Astley C., Souza D., Polito M. (2017). Acute caffeine ingestion on performance in young judo athletes. Pediatr. Exerc. Sci..

[46] Athayde M., Kons R., Detanico D. (2018). Can caffeine intake improve neuromuscular and technical-tactical performance during judo matches?. J. Strength Cond. Res..

[47] Carmo K.E.O., Pérez D.I.V., Valido C.N., Dos Santos J.L., Miarka B., Mendes-Netto R.S., Leite M.M.R., Antoniêtto N.R., Aedo-Muñoz E.A., Brito C.J. (2021). Caffeine improves biochemical and specific performance after judo training: A double-blind crossover study in a real judo training situation. Nutr. Metab..

[48] Diaz-Lara F.J., Del Coso J., García J.M., Portillo L.J., Areces F., Abián-Vicén J. (2016). Caffeine improves muscular performance in elite Brazilian Jiu-jitsu athletes. Eur. J. Sport Sci..

[49] Diaz-Lara F.J., Del Coso J., Portillo J., Areces F., García J.M., Abián-Vicén J. (2016). Enhancement of high-intensity actions and physical performance during a simulated Brazilian Jiu-Jitsu competition with a moderate dose of caffeine. Int. J. Sports Physiol. Perform..

[50] Durkalec-Michalski K., Nowaczyk P.M., Główka N., Grygiel A. (2019). Dose-dependent effect of caffeine supplementation on judo-specific performance and training activity: A randomized placebo-controlled crossover trial. J. Int. Soc. Sports Nutr..

[51] Filip-Stachnik A., Krawczyk R., Krzysztofik M., Rzeszutko-Belzowska A., Dornowski M., Zajac A., Del Coso J., Wilk M. (2021). Effects of acute ingestion of caffeinated chewing gum on performance in elite judo athletes. J. Int. Soc. Sports Nutr..

[52] Lopes-Silva J.P., Felippe L.J., Silva-Cavalcante M.D., Bertuzzi R., Lima-Silva A.E. (2014). Caffeine ingestion after rapid weight loss in judo athletes reduces perceived effort and increases plasma lactate concentration without improving performance. Nutrients.

[53] Lopes-Silva J.P., Rocha A., Rocha J.C.C., Silva V., Correia-Oliveira C.R. (2021). Caffeine ingestion increases the upper-body intermittent dynamic strength endurance performance of combat sports athletes. Eur. J. Sport Sci..

[54] Negaresh R., Del Coso J., Mokhtarzade M., Lima-Silva A.E., Baker J.S., Willems M.E.T., Talebvand S., Khodadoost M., Farhani F. (2019). Effects of different dosages of caffeine administration on wrestling performance during a simulated tournament. Eur. J. Sport Sci..

[55] Pereira L.A., Cyrino E.S., Avelar A., Segantin A.Q., Altimari J.M., Trindade M.C.d.C., Altimari L.R. (2010). A ingestão de cafeína não melhora o desempenho de atletas de judô. Motriz Rev. Educ. Fís..

[56] Athayde M., Kons R.L., Detanico D. (2019). An exploratory double-blind study of caffeine effects on performance and perceived exertion in Judo. Percept. Mot. Skills.

[57] Merino-Fernández M., Giráldez-Costas V., González-García J., Gutiérrez-Hellín J., González-Millán C., Matos-Duarte M., Ruiz-Moreno C. (2022). Effects of 3 mg/kg body mass of caffeine on the performance of Jiu-Jitsu elite athletes. Nutrients.

[58] Felippe L.C., Lopes-Silva J.P., Bertuzzi R., McGinley C., Lima-Silva A.E. (2016). Separate and combined effects of caffeine and sodium-bicarbonate intake on judo performance. Int. J. Sports Physiol. Perform..

[59] Krawczyk R., Krzysztofik M., Kostrzewa M., Komarek Z., Wilk M., Del Coso J., Filip-Stachnik A. (2022). Preliminary research towards acute effects of different doses of caffeine on strength-power performance in highly trained judo athletes. Int. J. Environ. Res. Public Health.

[60] Merino Fernandez M., Ruiz-Moreno C., Giraldez-Costas V., Gonzalez-Millan C., Matos-Duarte M., Gutierrez-Hellin J., Gonzalez-Garcia J. (2021). Caffeine Doses of 3 mg/kg increase unilateral and bilateral vertical jump outcomes in elite traditional Jiu-Jitsu athletes. Nutrients.

[61] Cortez L., Mackay K., Contreras E., Penailillo L. (2017). Acute effect of caffeine ingestion on reaction time and electromyographic activity of the Dollyo Chagi round kick in taekwondo fighters. RICYDE Rev. Int. Cienc. Deporte.

[62] Coswig V.S., Gentil P., Irigon F., Del Vecchio F.B. (2018). Caffeine ingestion changes time-motion and technical-tactical aspects in simulated boxing matches: A randomized double-blind PLA-controlled crossover study. Eur. J. Sport Sci..

[63] Rezaei S., Akbari K., Gahreman D.E., Sarshin A., Tabben M., Kaviani M., Sadeghinikoo A., Koozehchian M.S., Naderi A. (2019). Caffeine and sodium bicarbonate supplementation alone or together improve karate performance. J. Int. Soc. Sports Nutr..

[64] San Juan A.F., López-Samanes Á., Jodra P., Valenzuela P.L., Rueda J., Veiga-Herreros P., Pérez-López A., Domínguez R. (2019). Caffeine supplementation improves anaerobic performance and neuromuscular efficiency and fatigue in olympic-level boxers. Nutrients.

[65] Arazi H., Hoseinihaji M., Eghbali E. (2016). The effects of different doses of caffeine on performance, rating of perceived exertion and pain perception in teenagers female karate athletes. Braz. J. Pharm. Sci..

[66] Lopes-Silva J.P., Silva Santos J.F., Branco B.H., Abad C.C., Oliveira L.F., Loturco I., Franchini E. (2015). Caffeine ingestion increases estimated glycolytic metabolism during taekwondo combat simulation but does not improve performance or parasympathetic reactivation. PLoS ONE.

[67] Santos V.G., Santos V.R., Felippe L.J., Almeida J.W., Bertuzzi R., Kiss M.A., Lima-Silva A.E. (2014). Caffeine reduces reaction time and improves performance in simulated-contest of taekwondo. Nutrients.

[68] Ouergui I., Mahdi N., Delleli S., Messaoudi H., Chtourou H., Sahnoun Z., Bouassida A., Bouhlel E., Nobari H., Ardigò L.P. (2022). Acute effects of low dose of caffeine ingestion combined with conditioning activity on psychological and physical performances of male and female taekwondo athletes. Nutrients.

[69] De Azevedo A.P., Guerra M.A., Caldas L.C., Guimaraes-Ferreira L. (2019). Acute caffeine ingestion did not enhance punch performance in professional mixed-martial arts athletes. Nutrients.

[70] De Morton N.A. (2009). The PEDro scale is a valid measure of the methodological quality of clinical trials: A demographic study. Aust. J. Physiother..

[71] Maher C.G., Sherrington C., Herbert R.D., Moseley A.M., Elkins M. (2003). Reliability of the PEDro scale for rating quality of randomized controlled trials. Phys. Ther..

[72] Mielgo-Ayuso J., Marques-Jiménez D., Refoyo I., Del Coso J., León-Guereño P., Calleja-González J. (2019). Effect of caffeine supplementation on sports performance based on differences between sexes: A systematic review. Nutrients.

[73] Follmann D., Elliott P., Suh I., Cutler J. (1992). Variance imputation for overviews of clinical trials with continuous response. J. Clin. Epidemiol..

[74] Cohen J. (2013). Statistical Power Analysis for the Behavioral Sciences.

[75] Deeks J.J., Higgins J.P., Altman D.G., Group C.S.M. (2019). Analysing data and undertaking meta-analyses. Cochrane Handbook for Systematic Reviews of Interventions.

[76] Kontopantelis E., Springate D.A., Reeves D. (2013). A re-analysis of the Cochrane Library data: The dangers of unobserved heterogeneity in meta-analyses. PLoS ONE.

[77] Hedges L.V., Olkin I. (2014). Statistical Methods for Meta-Analysis.

[78] Begg C.B., Mazumdar M. (1994). Operating characteristics of a rank correlation test for publication bias. Biometrics.

[79] Egger M., Smith G.D., Schneider M., Minder C.J.B. (1997). Bias in meta-analysis detected by a simple, graphical test. BMJ.

[80] Duval S., Tweedie R. (2000). Trim and fill: A simple funnel-plot—based method of testing and adjusting for publication bias in meta-analysis. Biometrics.

[81] Higgins J., Green S. (2011). Cochrane Handbook for Systematic Reviews of Interventions.

[82] Kulinskaya E., Koricheva J. (2010). Use of quality control charts for detection of outliers and temporal trends in cumulative meta-analysis. Res. Synth. Methods.

[83] Ferreira R.E.S., Pacheco R.L., de Oliveira Cruz Latorraca C., Riera R., Eid R.G., Martimbianco A.L.C. (2021). Effects of caffeine supplementation on physical performance of soccer players: Systematic review and meta-analysis. Sports Health.

[84] Doherty M., Smith P.M. (2004). Effects of caffeine ingestion on exercise testing: A meta-analysis. Int. J. Sport Nutr. Exerc. Metab..

[85] Turnes T., Silva B.A., Kons R.L., Detanico D. (2022). Is bilateral deficit in handgrip strength associated with performance in specific judo tasks?. J. Strength Cond Res..

[86] Suchomel T.J., Nimphius S., Bellon C.R., Stone M.H. (2018). The importance of muscular strength: Training considerations. Sports Med..

[87] Ouergui I., Ardigò L.P., Selmi O., Levitt D.E., Chtourou H., Bouassida A., Bouhlel E., Franchini E. (2020). Changes in perceived exertion, well-being, and recovery during specific judo training: Impact of training period and exercise modality. Front. Physiol..

[88] Chen H.Y., Chen Y.C., Tung K., Chao H.H., Wang H.S. (2019). Effects of caffeine and sex on muscle performance and delayed-onset muscle soreness after exercise-induced muscle damage: A double-blind randomized trial. J. Appl. Physiol..

[89] Dominguez R., Veiga-Herreros P., Sanchez-Oliver A.J., Montoya J.J., Ramos-Alvarez J.J., Miguel-Tobal F., Lago-Rodriguez A., Jodra P. (2021). Acute effects of caffeine intake on psychological responses and high-intensity exercise performance. Int. J. Environ. Res. Public Health.

[90] Del Coso J., Salinero J.J., Gonzalez-Millan C., Abian-Vicen J., Perez-Gonzalez B. (2012). Dose response effects of a caffeine-containing energy drink on muscle performance: A repeated measures design. J. Int. Soc. Sports Nutr..

[91] Franchini E., Miarka B., Matheus L., Del Vecchio F.B. (2011). Endurance in judogi grip strength tests: Comparison between elite and non-elite judo players. Arch. Budo.

[92] Thibordee S., Prasartwuth O. (2014). Effectiveness of roundhouse kick in elite Taekwondo athletes. J. Electromyogr. Kinesiol..

[93] Bishop D. (2010). Dietary supplements and team-sport performance. Sports Med..

[94] Gladden L.B. (2008). A lactatic perspective on metabolism. Med. Sci. Sports Exerc..

[95] Mohr M., Nielsen J.J., Bangsbo J. (2011). Caffeine intake improves intense intermittent exercise performance and reduces muscle interstitial potassium accumulation. J. Appl Physiol.

[96] Lee C.L., Cheng C.F., Lin J.C., Huang H.W. (2012). Caffeine’s effect on intermittent sprint cycling performance with different rest intervals. Eur. J. Appl. Physiol.

[97] Buchheit M. (2014). Monitoring training status with HR measures: Do all roads lead to Rome?. Front. Physiol..

[98] Costin A., Costin N., Cohen P., Eisenach C., Marchlinski F. (2013). Effect of exercise on heart-rate response to mental stress in teenagers. Eur. J. Prev. Cardiol..

[99] Petzer A., Pienaar A., Petzer J.P. (2013). The interactions of caffeine with monoamine oxidase. Life Sci..

[100] Nurminen M.L., Niittynen L., Korpela R., Vapaatalo H. (1999). Coffee, caffeine and blood pressure: A critical review. Eur. J. Clin. Nutr..

[101] Benjamim C.J.R., Monteiro L.R.L., Pontes Y.M.M., Silva A., Souza T.K.M., Valenti V.E., Garner D.M., Cavalcante T.C.F. (2021). Caffeine slows heart rate autonomic recovery following strength exercise in healthy subjects. Rev. Port. Cardiol..

[102] Grgic J., Mikulic P. (2017). Caffeine ingestion acutely enhances muscular strength and power but not muscular endurance in resistance-trained men. Eur. J. Sport Sci..

[103] Rooks C.R., Thom N.J., McCully K.K., Dishman R.K. (2010). Effects of incremental exercise on cerebral oxygenation measured by near-infrared spectroscopy: A systematic review. Prog. Neurobiol..

[104] McCall A.L., Millington W.R., Wurtman R.J. (1982). Blood-brain barrier transport of caffeine: Dose-related restriction of adenine transport. Life Sci..

[105] Grgic J., Mikulic P., Schoenfeld B.J., Bishop D.J., Pedisic Z. (2019). The influence of caffeine supplementation on resistance exercise: A review. Sports Med..

[106] Pethick J., Winter S.L., Burnley M. (2017). Caffeine ingestion attenuates fatigue-induced loss of muscle torque complexity. Med. Sci. Sports Exerc..

[107] Borg G. (1970). Perceived exertion as an indicator of somatic stress. Scand. J. Rehabil. Med..

[108] Gonzaga L.A., Vanderlei L.C.M., Gomes R.L., Valenti V.E. (2017). Caffeine affects autonomic control of heart rate and blood pressure recovery after aerobic exercise in young adults: A crossover study. Sci. Rep..

[109] Miarka B., Fukuda H.D., Del Vecchio F.B., Franchini E. (2016). Discriminant analysis of technical-tactical actions in high-level judo athletes. Int. J. Perfor. Anal. Sport.

[110] Chaabene H., Hachana Y., Franchini E., Mkaouer B., Chamari K. (2012). Physical and physiological profile of elite karate athletes. Sports Med..

[111] Jacobson B.H., Hester G.M., Palmer T.B., Williams K., Pope Z.K., Sellers J.H., Conchola E.C., Woolsey C., Estrada C. (2018). Effect of Energy drink consumption on power and velocity of selected sport performance activities. J. Strength Cond. Res..

[112] Owens D.S. (2019). Lifestyle modification: Diet, exercise, sports, and other issues. Hypertrophic Cardiomyopathy.

[113] Pickering C. (2019). Caffeine, CYP1A2 genotype, and sports performance: Is timing important?. Ir. J. Med. Sci..

[114] Pickering C., Kiely J. (2019). What should we do about habitual caffeine use in athletes?. Sports Med..

[115] Bell D.G., McLellan T.M. (2002). Exercise endurance 1, 3, and 6 h after caffeine ingestion in caffeine users and nonusers. J. Appl. Physiol..

[116] Fredholm B.B., Bättig K., Holmén J., Nehlig A., Zvartau E.E. (1999). Actions of caffeine in the brain with special reference to factors that contribute to its widespread use. Pharmacology.

[117] Goncalves L.d.S., Painelli V.d.S., Yamaguchi G., Oliveira L.F.d., Saunders B., da Silva R.P., Maciel E., Artioli G.G., Roschel H., Gualano B. (2017). Dispelling the myth that habitual caffeine consumption influences the performance response to acute caffeine supplementation. J. Appl. Physiol..

[118] De Salles Painelli V., Teixeira E.L., Tardone B., Moreno M., Morandini J., Larrain V.H., Pires F.O. (2021). Habitual caffeine consumption does not interfere with the acute caffeine supplementation effects on strength endurance and jumping performance in trained individuals. Int. J. Sport Nutr. Exerc. Metab..

[119] Ruiz-Moreno C., Lara B., Salinero J.J., Brito de Souza D., Ordovas J.M., Del Coso J. (2020). Time course of tolerance to adverse effects associated with the ingestion of a moderate dose of caffeine. Eur. J. Nutr..

[120] Grgic J. (2021). Effects of caffeine on resistance exercise: A review of recent research. Sports Med..

[121] Hudson G.M., Green J.M., Bishop P.A., Richardson M.T. (2008). Effects of caffeine and aspirin on light resistance training performance, perceived exertion, and pain perception. J. Strength Cond. Res..

[122] Pickering C., Grgic J. (2019). Caffeine and exercise: What next?. Sports Med..

[123] Berthelot G., Sedeaud A., Marck A., Antero-Jacquemin J., Schipman J., Sauliere G., Marc A., Desgorces F.-D., Toussaint J.-F. (2015). Has athletic performance reached its peak?. Sports Med..

[124] Burke L.M. (2008). Caffeine and sports performance. Appl. Physiol. Nutr. Metab..

[125] Mizuno M., Kimura Y., Tokizawa K., Ishii K., Oda K., Sasaki T., Nakamura Y., Muraoka I., Ishiwata K. (2005). Greater adenosine A2A receptor densities in cardiac and skeletal muscle in endurance-trained men: A [11C] TMSX PET study. Nucl. Med. Biol..

[126] Swift C., Tiplady B. (1988). The effects of age on the response to caffeine. Psychopharmacology.

[127] Sterkowicz-Przybycien K., Fukuda D.H., Franchini E. (2019). Meta-analysis to determine normative values for the special judo fitness test in male athletes: 20+ years of sport-specific data and the lasting legacy of Stanislaw Sterkowicz. Sports.

[128] Pickering C., Kiely J. (2018). Are the current guidelines on caffeine use in sport optimal for everyone? Inter-individual variation in caffeine ergogenicity, and a move towards personalised sports nutrition. Sports Med..

[129] Spriet L.L. (2014). Exercise and sport performance with low doses of caffeine. Sports Med..

[130] Duncan M.J., Stanley M., Parkhouse N., Cook K., Smith M. (2013). Acute caffeine ingestion enhances strength performance and reduces perceived exertion and muscle pain perception during resistance exercise. Eur. J. Sport Sci..

[131] Zhang B., Liu Y., Wang X., Deng Y., Zheng X. (2020). Cognition and brain activation in response to various doses of caffeine: A near-infrared spectroscopy study. Front. Psychol..

[132] Kamimori G.H., Karyekar C.S., Otterstetter R., Cox D.S., Balkin T.J., Belenky G.L., Eddington N.D. (2002). The rate of absorption and relative bioavailability of caffeine administered in chewing gum versus capsules to normal healthy volunteers. Int. J. Pharm..

[133] Boyett J.C., Giersch G.E.W., Womack C.J., Saunders M.J., Hughey C.A., Daley H.M., Luden N.D. (2016). Time of day and training status both impact the efficacy of caffeine for short duration cycling performance. Nutrients.

[134] Mora-Rodríguez R., Pallarés J.G., López-Gullón J.M., López-Samanes Á., Fernández-Elías V.E., Ortega J.F. (2015). Improvements on neuromuscular performance with caffeine ingestion depend on the time-of-day. J. Sci. Med. Sport.

[135] Souissi Y., Souissi M., Chtourou H. (2019). Effects of caffeine ingestion on the diurnal variation of cognitive and repeated high-intensity performances. Pharmacol. Biochem. Behav..

[136] Souissi M., Abedelmalek S., Chtourou H., Atheymen R., Hakim A., Sahnoun Z. (2012). Effects of morning caffeine’ ingestion on mood States, simple reaction time, and short-term maximal performance on elite judoists. Asian J. Sports Med..

[137] Souissi M., Abedelmalek S., Bou Dhiba D., Theodoros Nikolaidis P., Ben Awicha H., Chtourou H., Sahnoun Z. (2015). Morning caffeine ingestion increases cognitive function and short-term maximal performance in footballer players after partial sleep deprivation. Biol. Rhythm. Res..

[138] Souissi M., Chikh N., Affès H., Sahnoun Z. (2018). Caffeine reversal of sleep deprivation effects on alertness, mood and repeated sprint performances in physical education students. Biol. Rhythm. Res..

[139] Souissi M., Souissi Y., Mseddi E., Sahnoun Z. (2021). The effects of caffeine on the diurnal variation of the reaction time and short-term maximal performance after one night of sleep deprivation. Biol. Rhythm. Res..

[140] Lane J., Steege J., Rupp S., Kuhn C. (1992). Menstrual cycle effects on caffeine elimination in the human female. Eur. J. Clin. Pharmacol..

[141] Areta J., Irwin C., Desbrow B. (2017). Inaccuracies in caffeine intake quantification and other important limitations in recent publication by Gonçalves et al. J. Appl. Physiol..

[142] Carrillo J.A., Christensen M., Ramos S.I., Alm C., Dahl M.-L., Benítez J., Bertilsson L. (2000). Evaluation of caffeine as an in vivo probe for CYP1A2 using measurements in plasma, saliva, and urine. Ther. Drug Monit..

[143] Bühler E., Lachenmeier D., Schlegel K., Winkler G. (2014). Development of a tool to assess the caffeine intake among teenagers and young adults. Ernahr. Umsch..

[144] Filip A., Wilk M., Krzysztofik M., Del Coso J. (2020). Inconsistency in the ergogenic effect of caffeine in athletes who regularly consume caffeine: Is it due to the disparity in the criteria that defines habitual caffeine intake?. Nutrients.

[145] Cohen J. (1988). Stafisfical Power Analysis for the Behavioural Sciences.

[146] Paton C.D., Lowe T., Irvine A. (2010). Caffeinated chewing gum increases repeated sprint performance and augments increases in testosterone in competitive cyclists. Eur. J. Appl. Physiol..

