# Supplementary material for: Acute Effects of Caffeine Supplementation on Physical Performance, Physiological Responses, Perceived Exertion, and Technical-Tactical Skills in Combat Sports: A Systematic Review and Meta-Analysis
Source: Nutrients. 2022 Jul 21;14(14):2996. doi: 10.3390/nu14142996 (PMC9315598; doi:10.3390/nu14142996)
Supplement: Supplementary file 1 [file nutrients-14-02996-s001.zip › nutrients-1828788-supplementary.pdf]

nutrients

MDPI

Supplementary Material

Table S1. List of excluded studies in each level.

Exclusion level	Studies
Duplication	(Astley et al., 2017; M. Athayde et al., 2018; M. S. D. Athayde et al., 2018; Athayde et al., 2019; Carmo et al., 2021; Cortez et al., 2017; Coswig et al., 2018; Da Silva Athayde et al., 2018; de Azevedo, Guerra, Caldas, & Guimaraes-Ferreira, 2019; de Azevedo, Guerra, Caldas, & Guimarães-Ferreira, 2019; Diaz-Lara, Del Coso, Garcia, et al., 2016; Diaz-Lara, Del Coso, García, et al., 2016a, 2016b; Diaz-Lara, del Coso, Portillo, Areces, Garcia, et al., 2016; Diaz-Lara, Del Coso, Portillo, Areces, et al., 2016a, 2016b; Durkalec-Michalski, Nowaczyk, Glowka, et al., 2019; Durkalec-Michalski, Nowaczyk, Główka, et al., 2019; Felippe et al., 2016; Filip-Stachnik et al., 2021a, 2021b; Grgic et al., 2021a; Lopes-Silva et al., 2014; Lopes-Silva et al., 2022; Lopes-Silva, Santos, et al., 2015; Lopes-Silva, Silva Santos, et al., 2015; Lopez-Gonzalez et al., 2018a; López-González et al., 2018; Merino-Fernandez et al., 2022; Merino-Fernández et al., 2022; Merino Fernández et al., 2021; Ouergui et al., 2022; Pak İ et al., 2020; Rezaei et al., 2019; Saldanha da Silva Athayde et al., 2019a, 2019b; Santos et al., 2014; Simoncini et al., 2021b)
Title	(Candia-Lujan et al., 2014; Diaz-Manzano et al., 2018; Eliasson et al., 2018; Grgic et al., 2021b; Harty et al., 2019; Hemmersbach, 2008; Hoffman et al., 2014; Lee et al., 2014; Lieberman et al., 2014; Lopes-Silva et al., 2018; Lopez-Gonzalez et al., 2018b; Pak et al., 2020; Rocha et al., 2016; Simoncini et al., 2021a; Sterkowicz-Przybycien et al., 2019; Yamazaki et al., 2019)
Abstract	(Congeni & Miller, 2002; Lin et al., 2014; Lopes-Silva et al., 2020; Reale et al., 2017; Striegel et al., 2005)
Full text	(Balko et al., 2020; Vidal & Prado, 2020)

Table S2. Results of the Begg and Mazumdar's Rank Correlation Test and Egger's Linear Regression Test

	Begg and Mazumdar's Rank Correlation Test							Egger's Linear Regression Test					
	**Kendall's S statistic P-Q**	**Tau without continuity correction**	**Z**	**P**	**Tau with continuity correction**	**Z**	**P**	**Intercept**	**SE**	**95% confidence interval**	**t**	**df**	**p**
**CMJ**	1	0.048	0.15	0.88	0	0	1	-0.14	2.59	-6.80 to 6.51	0.06	5	0.96
**Handgrip strength**	2	0.20	0.49	0.62	0.10	0.24	0.81	-1.60	7.29	-24.80 to 21.61	0.22	3	0.84
**JGST**	-1	0.33	0.52	0.60	0	0	1	-3.77	6.06	-80.80 to 73.26	0.62	1	0.64
**SJFT throws**	10	0.36	1.24	0.22	0.32	1.11	0.27	3.64	2.75	-3.09 to 10.37	1.32	6	0.23
**SJFT index**	-7	-0.33	1.05	0.29	-0.29	0.90	0.37	-6.06	1.97	-11.12 to -0.99	3.07	5	0.03
**RPE-post anaerobic test**	-9	-0429	1.35	0.176	-0.38	1.20	0.23	-1.44	3.16	-9.56 to 6.68	0.46	5	0.67
**RPE-post combat**	-2	-0.33	0.679	0.496	-0.167	0.34	0.73	-55.69	24.36	-160.50 to 49.11	2.29	2	0.15
**Offensives actions (N)**	-8	-0.286	0.99	0.32	-0.25	0.87	0.39	-0.83	3.37	-9.08 to 7.40	0.25	6	0.81
**Offensives actions (s)**	0	0	0	1	0	0	1	7.80	27.34	-109.84 to 125.43	0.29	2	0.80
**[La] (anaerobic)**	6	0.60	1.47	0.14	0.50	1.22	0.22	6.19	1.01	2.98 to 9.41	6.13	3	0.009
**[La] (combats)**	9	0.60	1.69	0.09	0.53	1.50	0.13	5.39	1.17	2.13 to 8.65	4.60	4	0.01
**HR final**	-3	-0.2	0.56	0.57	-0.13	0.38	0.70	-0.46	2.72	-8.007 to 7.09	0.17	4	0.87
**HR 1min**	-5	-0.33	0.94	0.35	-0.27	0.75	0.45	-1.61	2.13	-7.51 to 4.30	0.76	4	0.49
**HR end-of fight**	6	1	2.04	0.04	0.83	1.70	0.09	27.94	11.85	-23.05 to 78.92	2.36	2	0.14

RPE: Rating of Perceived Exertion; HR: Heart Rate; [La]: Blood Lactate; CMJ: Countermovement jump; SJFT: Special Judo Fitness Test

.

Supplementary Figures

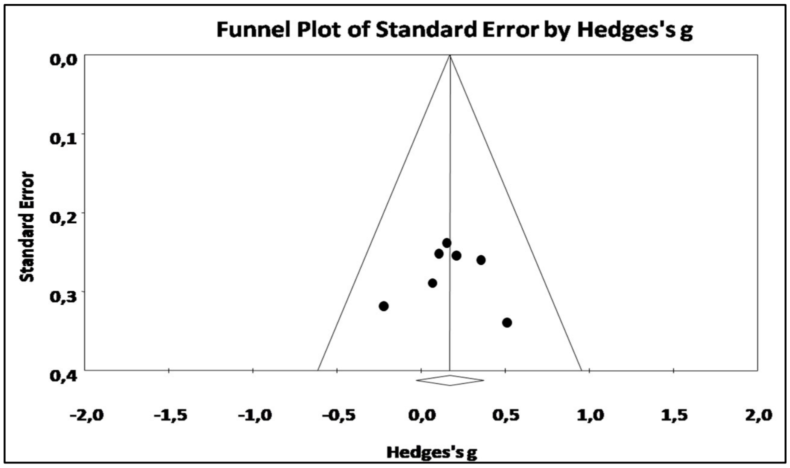

Figure S1. Funnel plot for CMJ showing no evidence of publication bias.

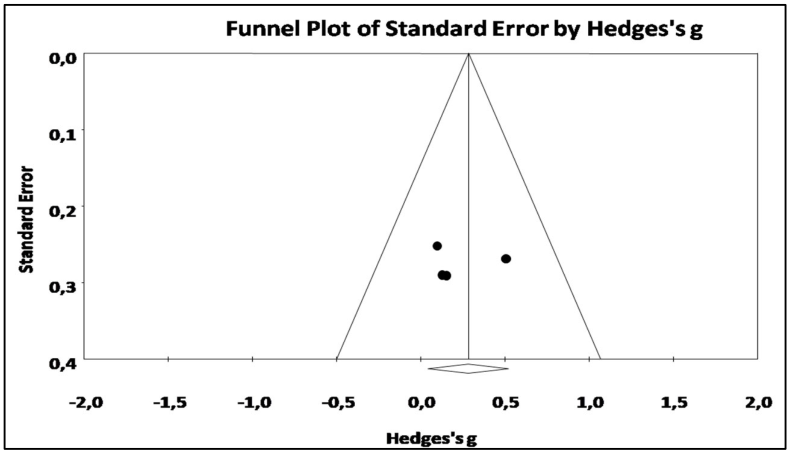

Figure S2. Funnel plot for handgrip strength showing no evidence of publication bias.

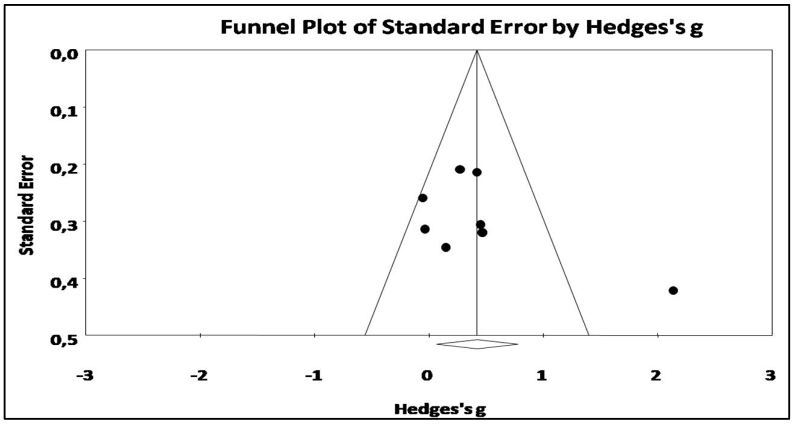

Figure S3. Funnel plot for SJFT number of throw showing no evidence of publication bias.

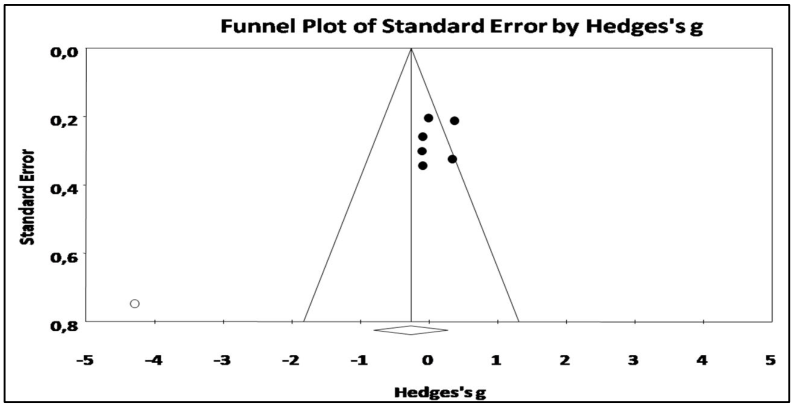

Figure S4. Funnel plot for SJFT index showing an evidence of publication bias.

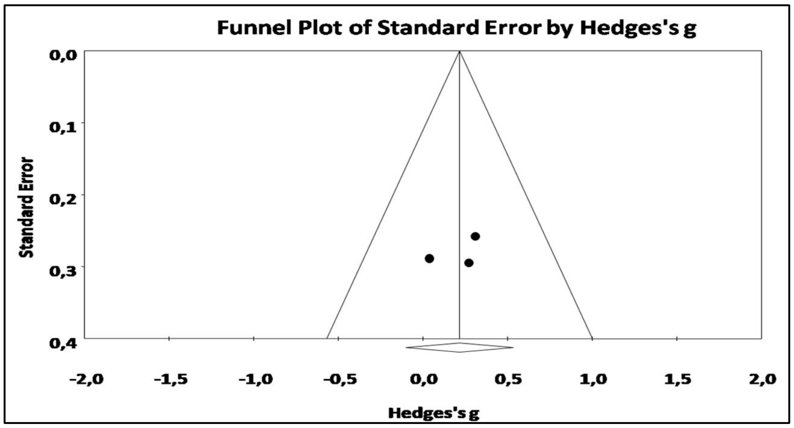

Figure S5. Funnel plot for judogi strength endurance test showing no evidence of publication bias.

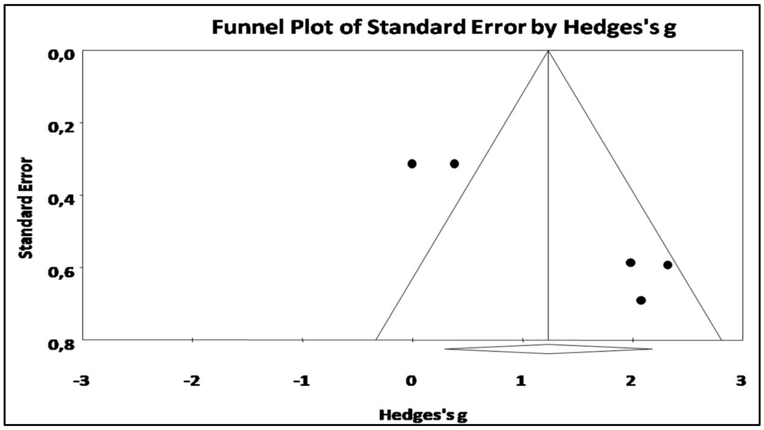

Figure S6. Funnel plot for [La] post-anaerobic exercise showing an evidence of publication bias.

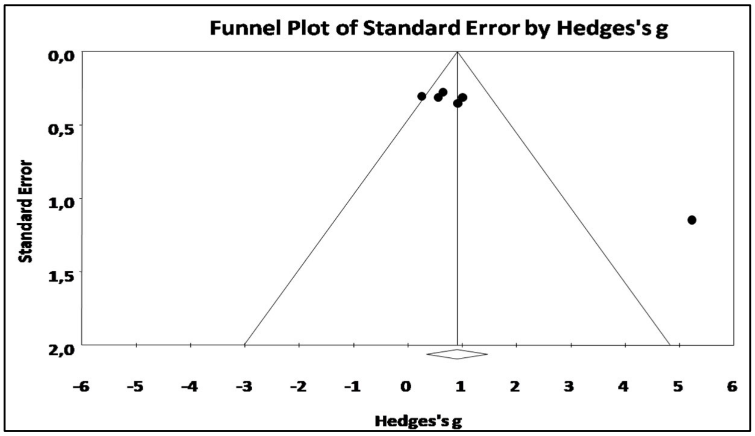

Figure S7. Funnel plot for [La] post-combat showing an evidence of publication bias.

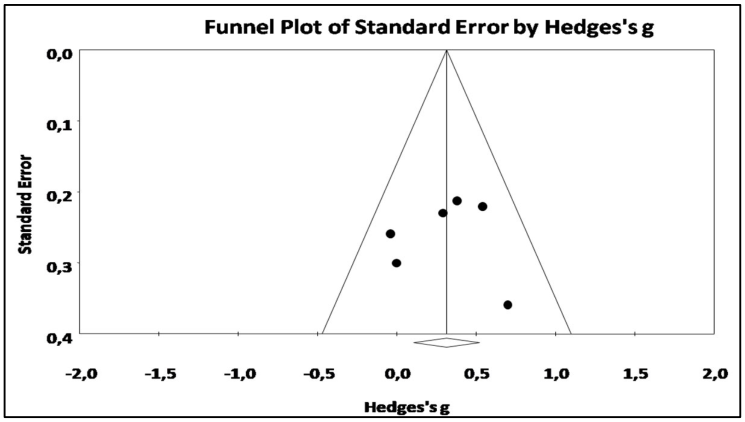

Figure S8. Funnel plot for HR final showing no evidence of publication bias.

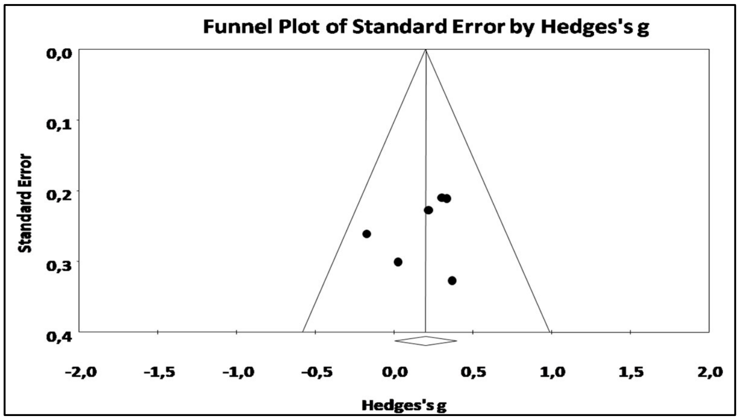

Figure S9. Funnel plot for HR 1min showing no evidence of publication bias.

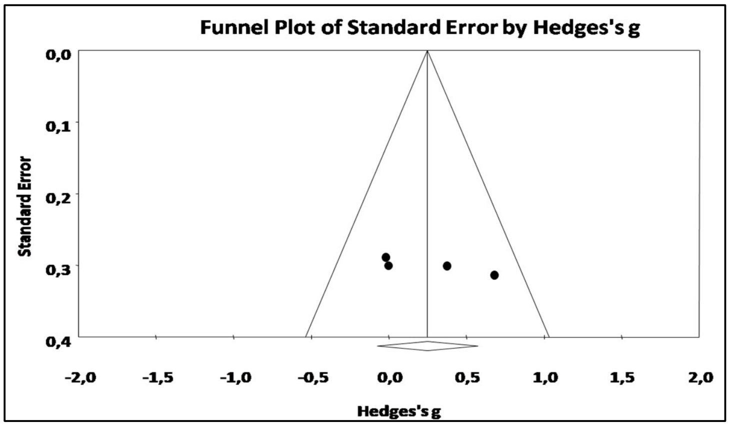

Figure S10. Funnel plot for HR post-combat showing no evidence of publication bias.

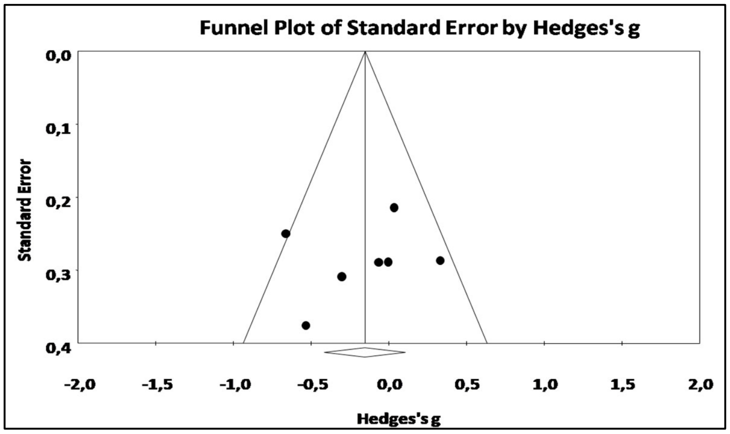

Figure S11. Funnel plot for RPE post-anaerobic test showing no evidence of publication bias.

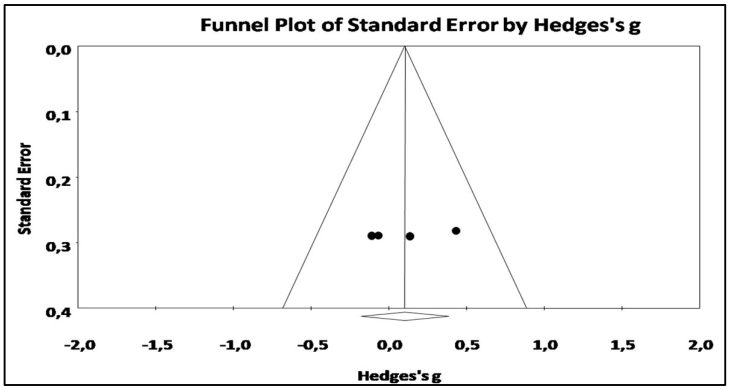

Figure S12. Funnel plot for RPE post-combat showing no evidence of publication bias.

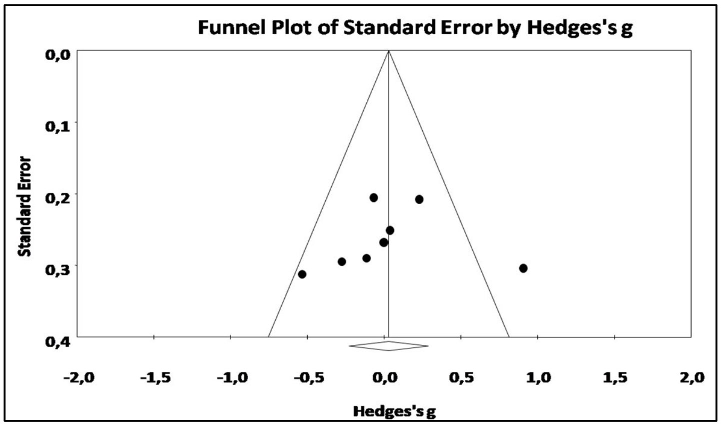

Figure S13. Funnel plot for the number of offensives actions showing no evidence of publication bias.

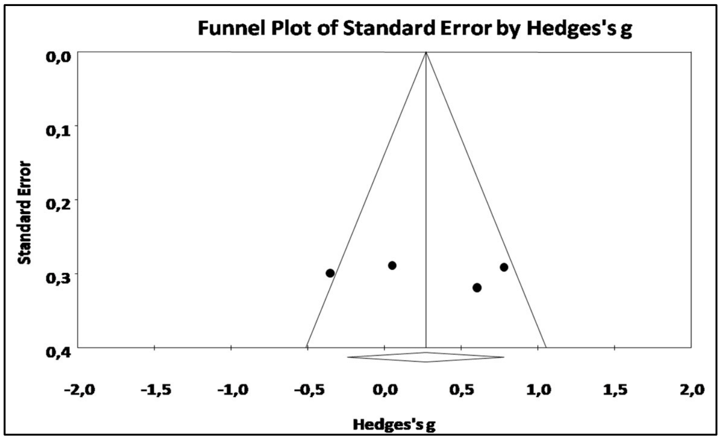

Figure S14 | Funnel plot for offensives actions duration showing no evidence of publication bias.

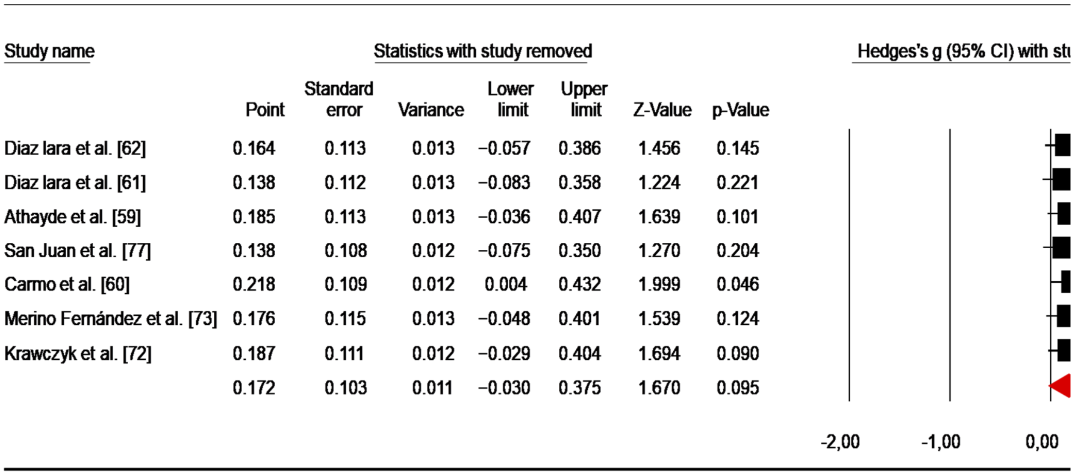

Study name	Point	Standard error	Variance	Lower limit	Upper limit	Z-Value	p-Value
	Statistics with study removed						
Diaz lara et al. [62]	0.164	0.113	0.013	−0.057	0.386	1.456	0.145
Diaz lara et al. [61]	0.138	0.112	0.013	−0.083	0.358	1.224	0.221
Athayde et al. [59]	0.185	0.113	0.013	−0.036	0.407	1.639	0.101
San Juan et al. [77]	0.138	0.108	0.012	−0.075	0.350	1.270	0.204
Carmo et al. [60]	0.218	0.109	0.012	0.004	0.432	1.999	0.046
Merino Fernández et al. [73]	0.176	0.115	0.013	−0.048	0.401	1.539	0.124
Krawczyk et al. [72]	0.187	0.111	0.012	−0.029	0.404	1.694	0.090
	0.172	0.103	0.011	−0.030	0.375	1.670	0.095

Figure S15. Forest plot of the leave-one-out sensitivity analysis for CMJ.

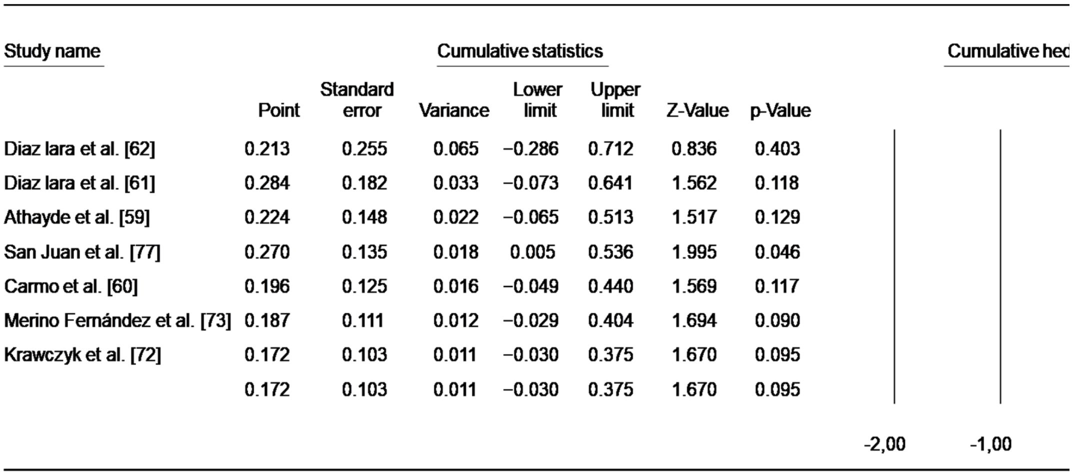

Study name	Point	Standard error	Variance	Lower limit	Upper limit	Z-Value	p-Value
	Cumulative statistics						
Diaz lara et al. [62]	0.213	0.255	0.065	−0.286	0.712	0.836	0.403
Diaz lara et al. [61]	0.284	0.182	0.033	−0.073	0.641	1.562	0.118
Athayde et al. [59]	0.224	0.148	0.022	−0.065	0.513	1.517	0.129
San Juan et al. [77]	0.270	0.135	0.018	0.005	0.536	1.995	0.046
Carmo et al. [60]	0.196	0.125	0.016	−0.049	0.440	1.569	0.117
Merino Fernández et al. [73]	0.187	0.111	0.012	−0.029	0.404	1.694	0.090
Krawczyk et al. [72]	0.172	0.103	0.011	−0.030	0.375	1.670	0.095
	0.172	0.103	0.011	−0.030	0.375	1.670	0.095

Figure S16. Forest plot of the cumulative meta-analysis for CMJ.

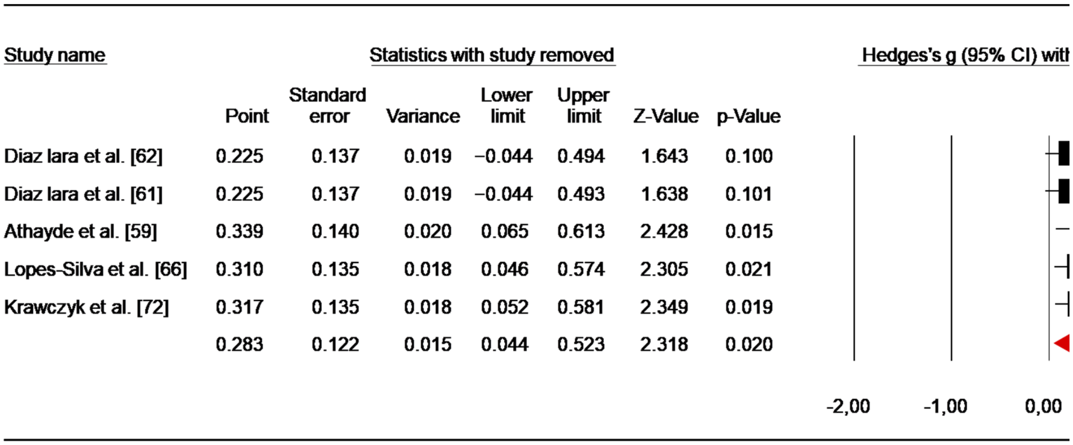

Study name	Point	Standard error	Variance	Lower limit	Upper limit	Z-Value	p-Value
	Statistics with study removed						
Diaz lara et al. [62]	0.225	0.137	0.019	−0.044	0.494	1.643	0.100
Diaz lara et al. [61]	0.225	0.137	0.019	−0.044	0.493	1.638	0.101
Athayde et al. [59]	0.339	0.140	0.020	0.065	0.613	2.428	0.015
Lopes-Silva et al. [66]	0.310	0.135	0.018	0.046	0.574	2.305	0.021
Krawczyk et al. [72]	0.317	0.135	0.018	0.052	0.581	2.349	0.019
	0.283	0.122	0.015	0.044	0.523	2.318	0.020

Figure S17. Forest plot of the leave-one-out sensitivity analysis for handgrip-strenght.

Study name	Cumulative statistics						
	Point	Standard error	Variance	Lower limit	Upper limit	Z-Value	p-Value
Diaz lara et al. [62]	0.506	0.269	0.072	−0.021	1.033	1.880	0.060
Diaz lara et al. [61]	0.508	0.190	0.036	0.135	0.881	2.667	0.008
Athayde et al. [59]	0.360	0.152	0.023	0.062	0.658	2.370	0.018
Lopes-Silva et al. [66]	0.317	0.135	0.018	0.052	0.581	2.349	0.019
Krawczyk et al. [72]	0.283	0.122	0.015	0.044	0.523	2.318	0.020
	0.283	0.122	0.015	0.044	0.523	2.318	0.020

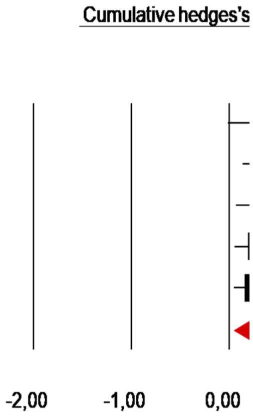

Figure S18. Forest plot of the cumulative meta-analysis for handgrip-strenght.

Study name	Statistics with study removed						
	Point	Standard error	Variance	Lower limit	Upper limit	Z-Value	p-Value
Pereira et al. [68]	0.497	0.200	0.040	0.104	0.889	2.478	0.013
Lopez Silva et al. [65]	0.460	0.204	0.042	0.061	0.860	2.257	0.024
Felippe et al. [71]	0.423	0.208	0.043	0.014	0.831	2.028	0.043
Astley et al. [58]	0.254	0.101	0.010	0.056	0.452	2.515	0.012
Durkalec-Michalski et al. [63]	0.457	0.221	0.049	0.023	0.890	2.065	0.039
Carmo et al. [60]	0.486	0.200	0.040	0.093	0.879	2.426	0.015
Filip-Stachnik et al. [64]	0.420	0.207	0.043	0.014	0.826	2.029	0.042
Merino-Fernández et al. [70]	0.432	0.221	0.049	−0.001	0.864	1.958	0.050
	0.421	0.183	0.033	0.063	0.779	2.305	0.021

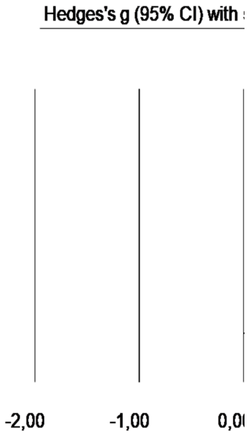

Figure S19. Forest plot of the leave-one-out sensitivity analysis for toatal number of throws during the SJFT.

Study name	Cumulative statistics						
	Point	Standard error	Variance	Lower limit	Upper limit	Z-Value	p-Value
Pereira et al. [68]	−0.050	0.260	0.068	−0.560	0.459	−0.194	0.846
Lopez Silva et al. [65]	0.022	0.208	0.043	−0.386	0.429	0.104	0.917
Felippe et al. [71]	0.158	0.172	0.030	−0.179	0.495	0.919	0.358
Astley et al. [58]	0.631	0.427	0.182	−0.206	1.468	1.479	0.139
Durkalec-Michalski et al. [63]	0.534	0.302	0.091	−0.059	1.127	1.766	0.077
Carmo et al. [60]	0.434	0.257	0.066	−0.071	0.938	1.684	0.092
Filip-Stachnik et al. (64]	0.432	0.221	0.049	−0.001	0.864	1.958	0.050
Merino-Fernández et al. [70]	0.421	0.183	0.033	0.063	0.779	2.305	0.021
	0.421	0.183	0.033	0.063	0.779	2.305	0.021

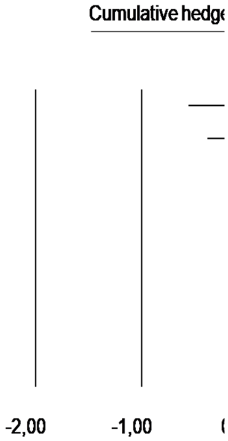

Figure S20. Forest plot of the cumulative meta-analysis for toatal number of throws during the SJFT.

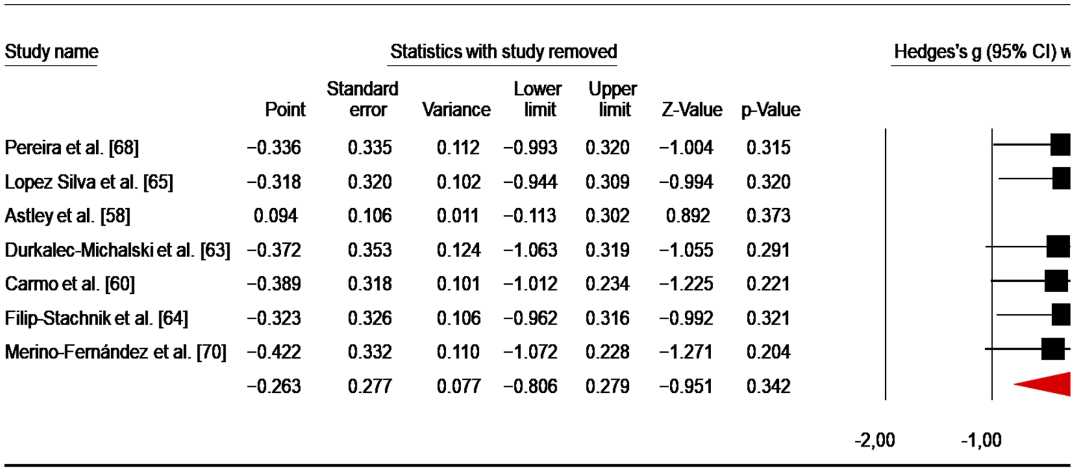

Study name	Point	Standard error	Variance	Lower limit	Upper limit	Z-Value	p-Value
Pereira et al. [68]	−0.336	0.335	0.112	−0.993	0.320	−1.004	0.315
Lopez Silva et al. [65]	−0.318	0.320	0.102	−0.944	0.309	−0.994	0.320
Astley et al. [58]	0.094	0.106	0.011	−0.113	0.302	0.892	0.373
Durkalec-Michalski et al. [63]	−0.372	0.353	0.124	−1.063	0.319	−1.055	0.291
Carmo et al. [60]	−0.389	0.318	0.101	−1.012	0.234	−1.225	0.221
Filip-Stachnik et al. [64]	−0.323	0.326	0.106	−0.962	0.316	−0.992	0.321
Merino-Fernández et al. [70]	−0.422	0.332	0.110	−1.072	0.228	−1.271	0.204
	−0.263	0.277	0.077	−0.806	0.279	−0.951	0.342

Figure S21. Forest plot of the leave-one-out sensitivity analysis for SJFT index.

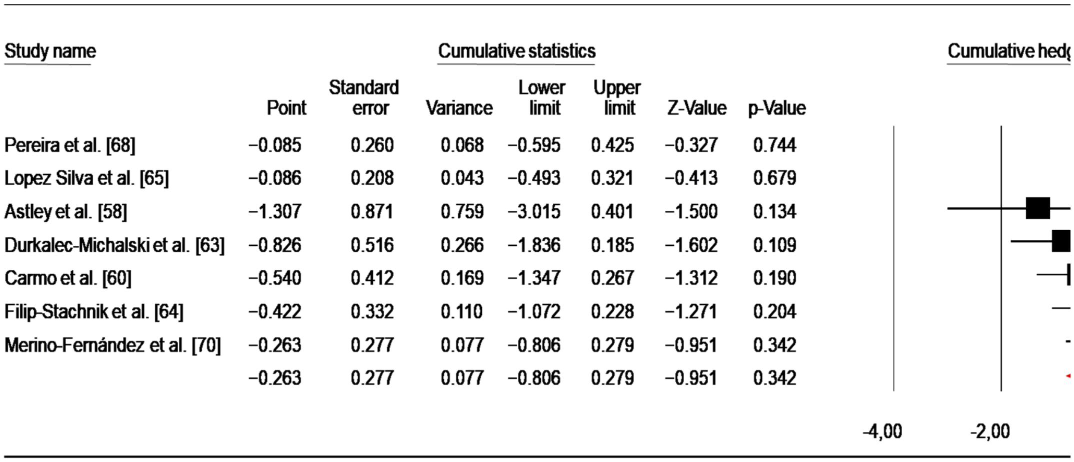

Study name	Point	Standard error	Variance	Lower limit	Upper limit	Z-Value	p-Value
Pereira et al. [68]	−0.085	0.260	0.068	−0.595	0.425	−0.327	0.744
Lopez Silva et al. [65]	−0.086	0.208	0.043	−0.493	0.321	−0.413	0.679
Astley et al. [58]	−1.307	0.871	0.759	−3.015	0.401	−1.500	0.134
Durkalec-Michalski et al. [63]	−0.826	0.516	0.266	−1.836	0.185	−1.602	0.109
Carmo et al. [60]	−0.540	0.412	0.169	−1.347	0.267	−1.312	0.190
Filip-Stachnik et al. [64]	−0.422	0.332	0.110	−1.072	0.228	−1.271	0.204
Merino-Fernández et al. [70]	−0.263	0.277	0.077	−0.806	0.279	−0.951	0.342
	−0.263	0.277	0.077	−0.806	0.279	−0.951	0.342

Figure S22. Forest plot of the cumulative meta-analysis for SJFT index.

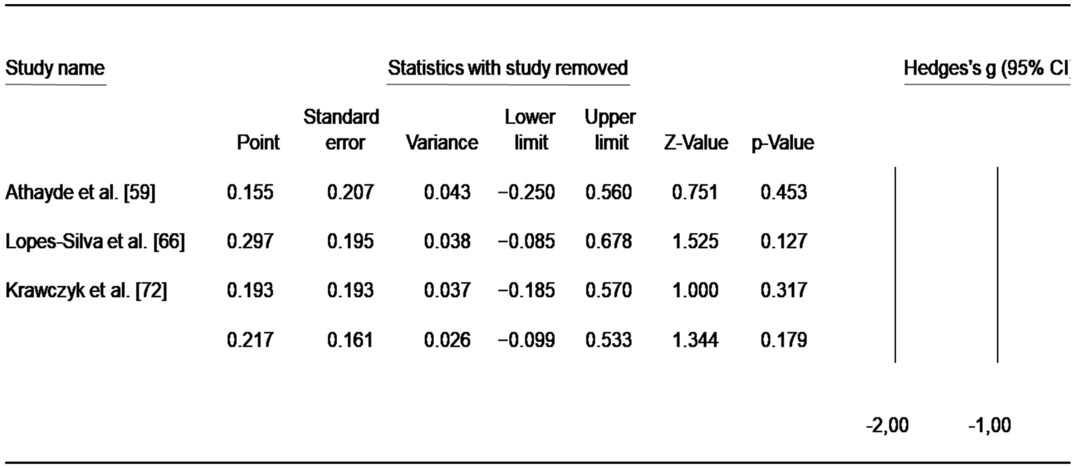

Study name	Point	Standard error	Variance	Lower limit	Upper limit	Z-Value	p-Value
Athayde et al. [59]	0.155	0.207	0.043	−0.250	0.560	0.751	0.453
Lopes-Silva et al. [66]	0.297	0.195	0.038	−0.085	0.678	1.525	0.127
Krawczyk et al. [72]	0.193	0.193	0.037	−0.185	0.570	1.000	0.317
	0.217	0.161	0.026	−0.099	0.533	1.344	0.179

Figure S23. Forest plot of the leave-one-out sensitivity analysis for the judogi strength endurance test.

Study name	Cumulative statistics						
	Point	Standard error	Variance	Lower limit	Upper limit	Z-Value	p-Value
Athayde et al. [59]	0.314	0.258	0.067	−0.193	0.820	1.214	0.225
Lopes-Silva et al. [66]	0.193	0.193	0.037	−0.185	0.570	1.000	0.317
Krawczyk et al. [72]	0.217	0.161	0.026	−0.099	0.533	1.344	0.179
	0.217	0.161	0.026	−0.099	0.533	1.344	0.179

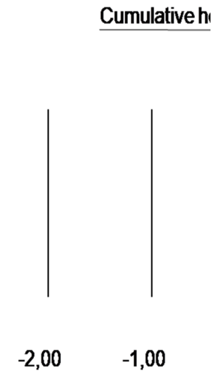

Figure S24. Forest plot of the cumulative meta-analysis for the judogi strength endurance test.

Study name	Statistics with study removed						
	Point	Standard error	Variance	Lower limit	Upper limit	Z-Value	p-Value
Lopes-Silva et al. [65]	1.058	0.517	0.268	0.045	2.072	2.046	0.041
Felippe et al. [71]	0.957	0.474	0.225	0.027	1.887	2.017	0.044
San Juan et al. [77]	1.056	0.521	0.271	0.035	2.076	2.028	0.043
Carmo et al. [60]	1.608	0.562	0.315	0.507	2.709	2.863	0.004
Filip-Stachnik et al. [64]	1.528	0.682	0.465	0.192	2.865	2.241	0.025
	1.233	0.482	0.232	0.289	2.177	2.560	0.010

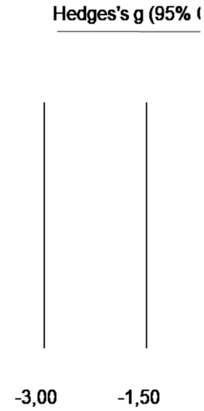

Figure S25. Forest plot of the leave-one-out sensitivity analysis for [La] post-anaerobic exercise.

Study name	Cumulative statistics						
	Point	Standard error	Variance	Lower limit	Upper limit	Z-Value	p-Value
Lopes-Silva et al. [65]	2.078	0.691	0.478	0.723	3.433	3.005	0.003
Felippe et al. [71]	2.217	0.450	0.203	1.334	3.100	4.921	0.000
San Juan et al. [77]	2.130	0.357	0.128	1.430	2.830	5.961	0.000
Carmo et al. [60]	1.528	0.682	0.465	0.192	2.865	2.241	0.025
Filip-Stachnik et al. [64]	1.233	0.482	0.232	0.289	2.177	2.560	0.010
	1.233	0.482	0.232	0.289	2.177	2.560	0.010

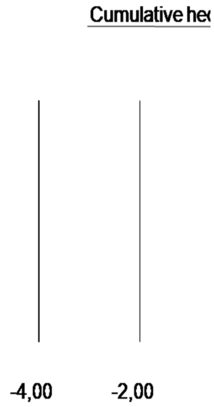

Figure S26. Forest plot of the cumulative meta-analysis for [La] post-anaerobic exercise.

Study name	Statistics with study removed							Hedges's g (95%
	Point	Standard error	Variance	Lower limit	Upper limit	Z-Value	p-Value	
Santos et al. [80]	1.040	0.362	0.131	0.331	1.750	2.874	0.004	
Lopes-Silva et al. [79]	0.947	0.353	0.124	0.255	1.638	2.684	0.007	
Diaz-Lara, et al. [62]	0.928	0.356	0.127	0.230	1.626	2.607	0.009	
Athayde et al. [59]	1.036	0.375	0.140	0.302	1.771	2.765	0.006	
Negaresh et al. [67]	0.666	0.140	0.019	0.393	0.940	4.774	0.000	
Filip-Stachnik et al. [64]	1.081	0.338	0.114	0.418	1.745	3.196	0.001	
	0.906	0.288	0.083	0.341	1.471	3.145	0.002	

-2,00 -1,00

Figure S27. Forest plot of the leave-one-out sensitivity analysis for [La] post-combat.

Study name	Cumulative statistics							Cumulative hedg
	Point	Standard error	Variance	Lower limit	Upper limit	Z-Value	p-Value	
Santos et al. [80]	0.561	0.315	0.099	−0.057	1.179	1.780	0.075	
Lopes-Silva et al. [79]	0.721	0.236	0.056	0.259	1.183	3.057	0.002	
Diaz-Lara, et al. [62]	0.825	0.189	0.036	0.455	1.196	4.366	0.000	
Athayde et al. [59]	0.771	0.157	0.025	0.464	1.078	4.921	0.000	
Negaresh et al. [67]	1.081	0.338	0.114	0.418	1.745	3.196	0.001	
Filip-Stachnik et al. [64]	0.906	0.288	0.083	0.341	1.471	3.145	0.002	
	0.906	0.288	0.083	0.341	1.471	3.145	0.002	

-2,00 -1,00 0

Figure S28. Forest plot of the cumulative meta-analysis for [La] post-combat.

Study name	Statistics with study removed							Hedges's g (95% CI) witl
	Point	Standard error	Variance	Lower limit	Upper limit	Z-Value	p-Value	
Pereira et al. [68]	0.380	0.112	0.012	0.161	0.599	3.397	0.001	
Astley et al. [58]	0.315	0.134	0.018	0.053	0.576	2.358	0.018	
Durkalec-Michalski et al. [63]	0.291	0.134	0.018	0.028	0.554	2.167	0.030	
Carmo et al. [60]	0.280	0.107	0.011	0.070	0.490	2.612	0.009	
Filip-Stachnik et al. [64]	0.356	0.110	0.012	0.141	0.571	3.244	0.001	
Merino-Fernández et al. [70]	0.252	0.116	0.013	0.024	0.479	2.171	0.030	
	0.313	0.106	0.011	0.106	0.520	2.967	0.003	

-2,00 -1,00 0,

Figure S29. Forest plot of the leave-one-out sensitivity analysis for HR final

Study name	Cumulative statistics						
	Point	Standard error	Variance	Lower limit	Upper limit	Z-Value	p-Value
Pereira et al. [68]	−0.039	0.260	0.067	−0.548	0.470	−0.149	0.881
Astley et al. [58]	0.147	0.172	0.030	−0.191	0.484	0.851	0.395
Durkalec-Michalski et al. [63]	0.239	0.134	0.018	−0.024	0.502	1.784	0.074
Carmo et al. [60]	0.296	0.128	0.016	0.046	0.546	2.317	0.021
Filip-Stachnik et al. [64]	0.252	0.116	0.013	0.024	0.479	2.171	0.030
Merino-Fernández et al. [70]	0.313	0.106	0.011	0.106	0.520	2.967	0.003
	0.313	0.106	0.011	0.106	0.520	2.967	0.003

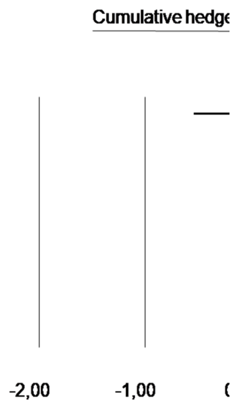

Figure S30. Forest plot of the cumulative meta-analysis for HR final.

Study name	Statistics with study removed						
	Point	Standard error	Variance	Lower limit	Upper limit	Z-Value	p-Value
Pereira et al. [68]	0.265	0.109	0.012	0.052	0.479	2.439	0.015
Astley et al. [58]	0.197	0.112	0.013	−0.023	0.416	1.756	0.079
Durkalec-Michalski et al. [63]	0.171	0.114	0.013	−0.053	0.395	1.493	0.135
Carmo et al. [60]	0.183	0.106	0.011	−0.024	0.390	1.737	0.082
Filip-Stachnik et al. [64]	0.223	0.107	0.011	0.014	0.432	2.092	0.036
Merino Fernández et al. [70]	0.161	0.114	0.013	−0.063	0.385	1.412	0.158
	0.201	0.101	0.010	0.004	0.398	2.002	0.045

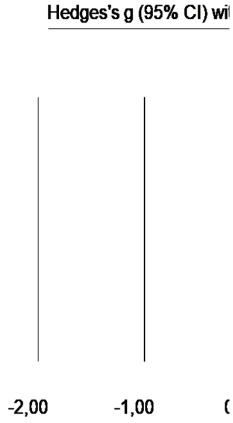

Figure S31. Forest plot of the leave-one-out sensitivity analysis for HR 1 min.

Study name	Cumulative statistics						
	Point	Standard error	Variance	Lower limit	Upper limit	Z-Value	p-Value
Pereira et al. [68]	−0.170	0.262	0.069	−0.684	0.343	−0.651	0.515
Astley et al. [58]	0.046	0.194	0.038	−0.335	0.426	0.237	0.813
Durkalec-Michalski et al., [63]	0.151	0.138	0.019	−0.119	0.421	1.095	0.273
Carmo et al. [60]	0.184	0.123	0.015	−0.058	0.426	1.489	0.136
Filip-Stachnik et al. [64]	0.161	0.114	0.013	−0.063	0.385	1.412	0.158
Merino Fernández et al. [70]	0.201	0.101	0.010	0.004	0.398	2.002	0.045
	0.201	0.101	0.010	0.004	0.398	2.002	0.045

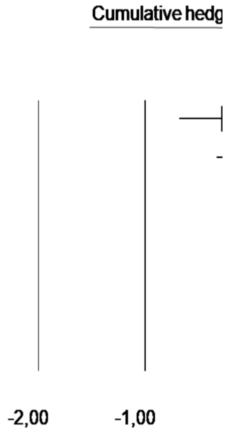

Figure S32. Forest plot of the cumulative meta-analysis for HR 1 min.

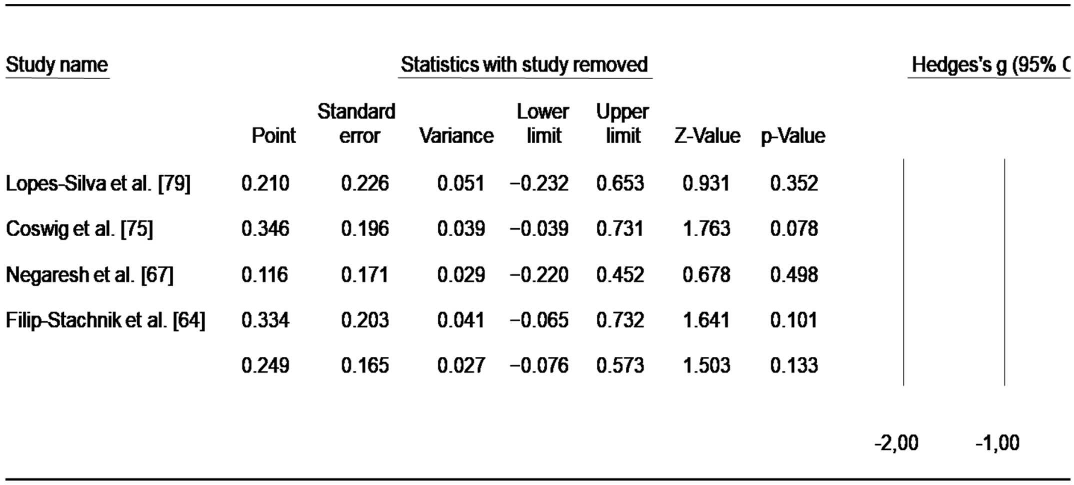

Figure S33. Forest plot of the leave-one-out sensitivity analysis for HR at the end-of-fight.

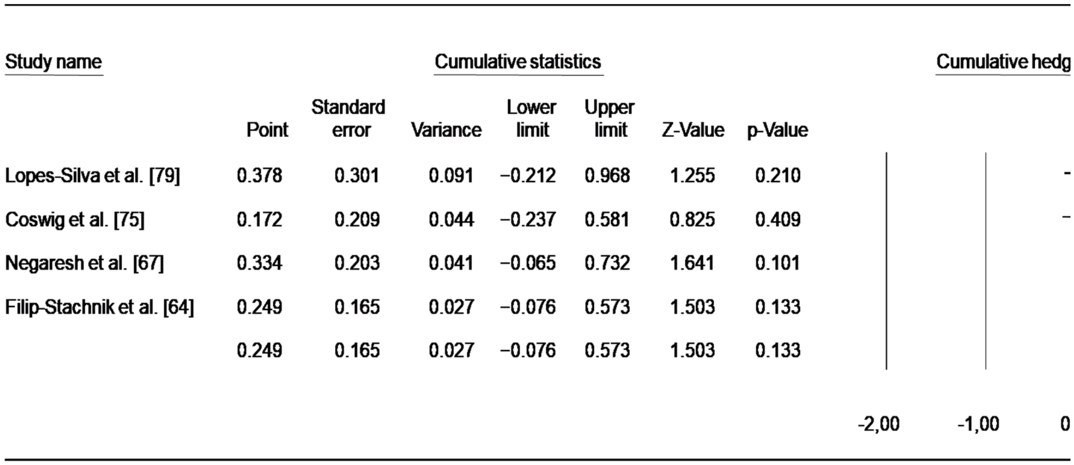

Figure S34. Forest plot of the cumulative meta-analysis for HR at the end-of-fight.

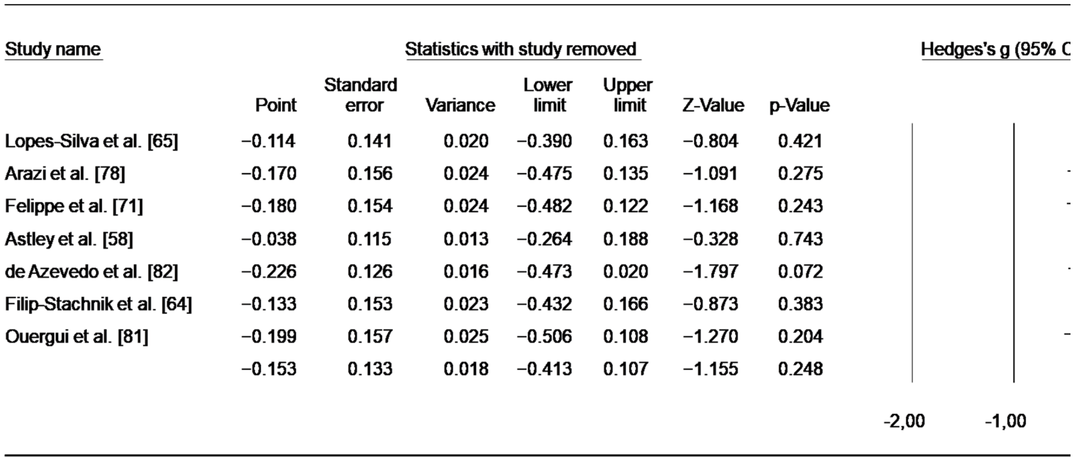

Figure S35. Forest plot of the leave-one-out sensitivity analysis for RPE post-anaerobic exercise.

Study name	Cumulative statistics						
	Point	Standard error	Variance	Lower limit	Upper limit	Z-Value	p-Value
Lopes-Silva et al. [65]	−0.530	0.376	0.142	−1.267	0.208	−1.408	0.159
Arazi et al. [78]	−0.237	0.229	0.053	−0.686	0.213	−1.031	0.302
Felippe et al. [71]	−0.145	0.180	0.032	−0.497	0.207	−0.808	0.419
Astley et al. [58]	−0.314	0.174	0.030	−0.656	0.027	−1.805	0.071
de Azevedo et al. [82]	−0.179	0.189	0.036	−0.550	0.192	−0.947	0.344
Filip-Stachnik et al. [64]	−0.199	0.157	0.025	−0.506	0.108	−1.270	0.204
Ouergui et al. [81]	−0.153	0.133	0.018	−0.413	0.107	−1.155	0.248
	−0.153	0.133	0.018	−0.413	0.107	−1.155	0.248

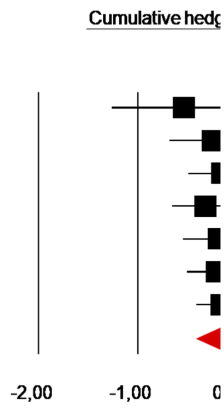

Figure S36. Forest plot of the cumulative meta-analysisfor RPE post-anaerobic exercise.

Study name	Statistics with study removed						
	Point	Standard error	Variance	Lower limit	Upper limit	Z-Value	p-Value
Santos et al. [80]	0.091	0.175	0.031	−0.253	0.434	0.518	0.605
Lopes-Silva et al. [79]	0.172	0.166	0.028	−0.153	0.498	1.037	0.300
Coswig et al. [75]	0.159	0.166	0.028	−0.167	0.485	0.957	0.339
Athayde et al. [69]	−0.013	0.168	0.028	−0.341	0.315	−0.077	0.939
	0.103	0.144	0.021	−0.180	0.385	0.714	0.475

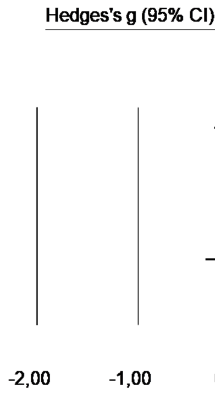

Figure S37. Forest plot of the leave-one-out sensitivity analysis for RPE post-combat.

Study name	Cumulative statistics						
	Point	Standard error	Variance	Lower limit	Upper limit	Z-Value	p-Value
Santos et al. [80]	0.138	0.291	0.085	−0.432	0.708	0.476	0.634
Lopes-Silva et al. [79]	0.015	0.205	0.042	−0.388	0.417	0.071	0.943
Coswig et al. [75]	−0.013	0.168	0.028	−0.341	0.315	−0.077	0.939
Athayde et al. [69]	0.103	0.144	0.021	−0.180	0.385	0.714	0.475
	0.103	0.144	0.021	−0.180	0.385	0.714	0.475

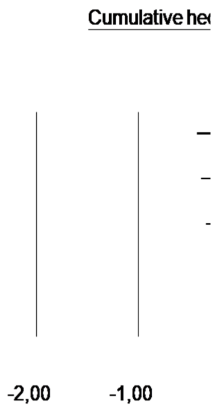

Figure S38. Forest plot of the cumulative meta-analysis for RPE post-combat.

Study name	Statistics with study removed						
	Point	Standard error	Variance	Lower limit	Upper limit	Z-Value	p-Value
Santos et al. [80]	0.047	0.148	0.022	−0.243	0.337	0.317	0.751
Lopes-Silva et al. [79]	0.067	0.142	0.020	−0.212	0.346	0.469	0.639
Diaz-Lara et al. [62]	−0.051	0.095	0.009	−0.238	0.136	−0.534	0.593
Athayde et al. [59]	0.027	0.152	0.023	−0.272	0.325	0.175	0.861
Coswig et al. [75]	0.093	0.127	0.016	−0.157	0.343	0.729	0.466
Durkalec et al. [63]	−0.007	0.151	0.023	−0.303	0.288	−0.049	0.961
Athayde et al. [69]	0.032	0.151	0.023	−0.263	0.328	0.214	0.830
Merino Fernendez et al. [70]	0.045	0.156	0.024	−0.260	0.350	0.290	0.771
	0.029	0.131	0.017	−0.228	0.286	0.222	0.824

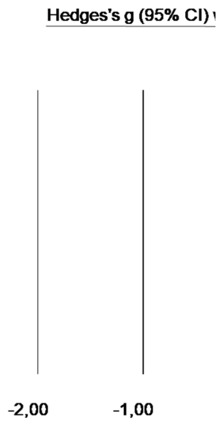

Figure S39. Forest plot of the leave-one-out sensitivity analysis for the number of offensives actions.

Study name	Cumulative statistics						
	Point	Standard error	Variance	Lower limit	Upper limit	Z-Value	p-Value
Santos et al. [80]	−0.113	0.290	0.084	−0.682	0.455	−0.391	0.696
Lopes-Silva et al. [79]	−0.192	0.207	0.043	−0.598	0.214	−0.927	0.354
Diaz-Lara et al. [62]	0.170	0.365	0.133	−0.546	0.886	0.466	0.641
Athayde et al. [59]	0.133	0.250	0.062	−0.356	0.622	0.534	0.594
Coswig et al. [75]	0.007	0.232	0.054	−0.449	0.462	0.028	0.978
Durkalec et al. [63]	0.051	0.184	0.034	−0.310	0.413	0.277	0.781
Athayde et al. [69]	0.045	0.156	0.024	−0.260	0.350	0.290	0.771
Merino Fernendez et al. [70]	0.029	0.131	0.017	−0.228	0.286	0.222	0.824
	0.029	0.131	0.017	−0.228	0.286	0.222	0.824

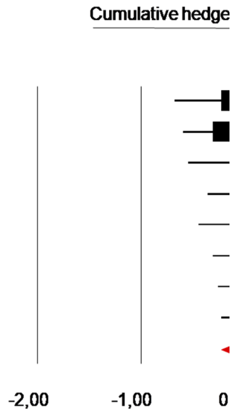

Figure S40. Forest plot of the cumulative meta-analysis for the number of offensives actions.

Study name	Statistics with study removed						
	Point	Standard error	Variance	Lower limit	Upper limit	Z-Value	p-Value
Santos et al. [80]	0.346	0.357	0.128	−0.354	1.046	0.968	0.333
Lopes-Silva et al. [79]	0.476	0.225	0.051	0.035	0.917	2.115	0.034
Diaz-Lara et al. [62]	0.095	0.271	0.073	−0.435	0.625	0.351	0.726
Coswig et al. [75]	0.165	0.330	0.109	−0.482	0.812	0.500	0.617
	0.271	0.260	0.068	−0.239	0.781	1.043	0.297

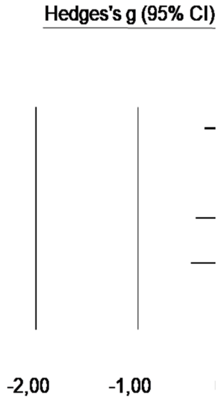

Figure S41. Forest plot of the leave-one-out sensitivity analysis for the duration of offensives actions.

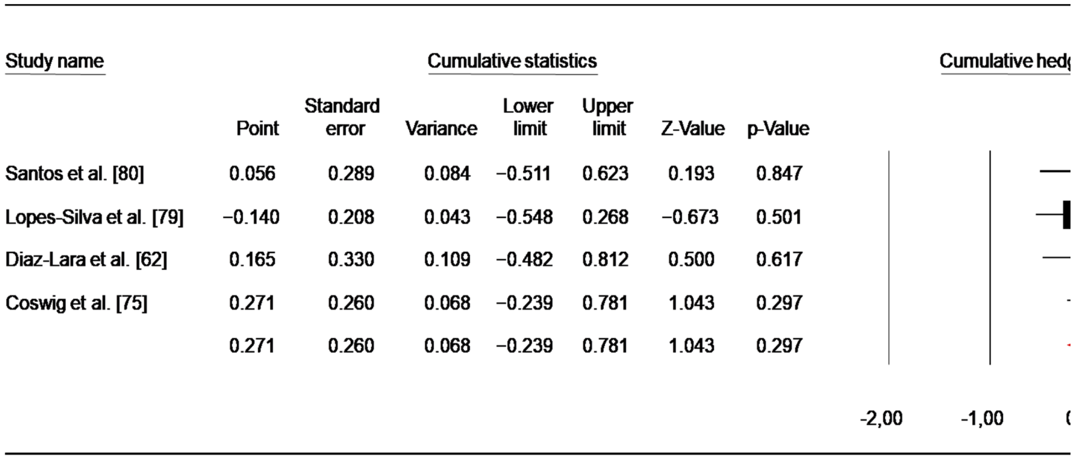

Figure S42. Forest plot of the cumulative meta-analysis for the duration of offensives actions.

PRISMA 2020 Main Checklist

Topic	No.	Item	Location where item is reported
**TITLE**			
**Title**	1	Identify the report as a systematic review.	Title (p.1)
**ABSTRACT**			
**Abstract**	2	See the PRISMA 2020 for Abstracts checklist	Abstract (p.1-2)
**INTRODUCTION**			
**Rationale**	3	Describe the rationale for the review in the context of existing knowledge.	Introduction (p. 2-4)
**Objectives**	4	Provide an explicit statement of the objective(s) or question(s) the review addresses.	Introduction (p.4)
**METHODS**			
**Eligibility criteria**	5	Specify the inclusion and exclusion criteria for the review and how studies were grouped for the syntheses.	Eligibility criteria(p.4)
**Information sources**	6	Specify all databases, registers, websites, organisations, reference lists and other sources searched or consulted to identify studies. Specify the date when each source was last searched or consulted.	Search strategy (p.5)
**Search strategy**	7	Present the full search strategies for all databases, registers and websites, including any filters and limits used.	Search strategy (p.5)

Topic	No.	Item	Location where item is reported
**Selection process**	8	Specify the methods used to decide whether a study met the inclusion criteria of the review, including how many reviewers screened each record and each report retrieved, whether they worked independently, and if applicable, details of automation tools used in the process.	Selection process (p.5-6)
**Data collection process**	9	Specify the methods used to collect data from reports, including how many reviewers collected data from each report, whether they worked independently, any processes for obtaining or confirming data from study investigators, and if applicable, details of automation tools used in the process.	Data extraction and coding (p.6)
**Data items**	10a	List and define all outcomes for which data were sought. Specify whether all results that were compatible with each outcome domain in each study were sought (e.g. for all measures, time points, analyses), and if not, the methods used to decide which results to collect.	Data extraction and coding (p.6)
	10b	List and define all other variables for which data were sought (e.g. participant and intervention characteristics, funding sources). Describe any assumptions made about any missing or unclear information.	Data extraction and coding (p.6)
**Study risk of bias assessment**	11	Specify the methods used to assess risk of bias in the included studies, including details of the tool(s) used, how many reviewers assessed each study and whether they worked independently, and if applicable, details of automation tools used in the process.	Risk of bias assessment (p.6-7)
**Effect measures**	12	Specify for each outcome the effect measure(s) (e.g. risk ratio, mean difference) used in the synthesis or presentation of results.	Data synthesis and analyses (p.7)
**Synthesis methods**	13a	Describe the processes used to decide which studies were eligible for each synthesis (e.g. tabulating the study intervention characteristics and comparing against the planned groups for each synthesis (item 5)).	Data synthesis and analyses (p.7-8)
	13b	Describe any methods required to prepare the data for presentation or synthesis, such as handling of missing summary statistics, or data conversions.	Data synthesis and analyses (p.7-8)
	13c	Describe any methods used to tabulate or visually display results of individual studies and syntheses.	Data synthesis and analyses (p.7-8)
	13d	Describe any methods used to synthesize results and provide a rationale for the choice(s). If meta-analysis was performed, describe the model(s), method(s) to identify the presence and extent of statistical heterogeneity, and software package(s) used.	Data synthesis and analyses (p.7-8)

Topic	No.	Item	Location where item is reported
	13e	Describe any methods used to explore possible causes of heterogeneity among study results (e.g. subgroup analysis, meta-regression).	Data synthesis and analyses (p.7-8)
	13f	Describe any sensitivity analyses conducted to assess robustness of the synthesized results.	Data synthesis and analyses (p.7-8)
**Reporting bias assessment**	14	Describe any methods used to assess risk of bias due to missing results in a synthesis (arising from reporting biases).	Data synthesis and analyses (p.8)
**Certainty assessment**	15	Describe any methods used to assess certainty (or confidence) in the body of evidence for an outcome.	Certainty was no assessed
**RESULTS**			
**Study selection**	16a	Describe the results of the search and selection process, from the number of records identified in the search to the number of studies included in the review, ideally using a flow diagram.	Search results (p.8)
	16b	Cite studies that might appear to meet the inclusion criteria, but which were excluded, and explain why they were excluded.	Search results (p.8)
**Study characteristics**	17	Cite each included study and present its characteristics.	Studies characteristics (p.8-10)
**Risk of bias in studies**	18	Present assessments of risk of bias for each included study.	Risk of bias assessment (p.11)
**Results of individual studies**	19	For all outcomes, present, for each study: (a) summary statistics for each group (where appropriate) and (b) an effect estimate and its precision (e.g. confidence/credible interval), ideally using structured tables or plots.	Meta-analysis results (p.11-17)
**Results of syntheses**	20a	For each synthesis, briefly summarise the characteristics and risk of bias among contributing studies.	Meta-analysis results (p.11-17)
	20b	Present results of all statistical syntheses conducted. If meta-analysis was done, present for each the summary estimate and its precision (e.g. confidence/credible interval) and measures of statistical heterogeneity. If comparing groups, describe the direction of the effect.	Meta-analysis results (p.11-17)
	20c	Present results of all investigations of possible causes of heterogeneity among study results.	Meta-analysis results (p.11-17)
	20d	Present results of all sensitivity analyses conducted to assess the robustness of the synthesized results.	Stability and reliability of the results (p.17)

Topic	No.	Item	Location where item is reported
**Reporting biases**	21	Present assessments of risk of bias due to missing results (arising from reporting biases) for each synthesis assessed.	Meta-analysis results (p.11-17)
**Certainty of evidence**	22	Present assessments of certainty (or confidence) in the body of evidence for each outcome assessed.	Certainty was no assessed
**DISCUSSION**			
**Discussion**	23a	Provide a general interpretation of the results in the context of other evidence.	Discussion (p.18-35)
	23b	Discuss any limitations of the evidence included in the review.	Methodological recommendations (p.35-37)
	23c	Discuss any limitations of the review processes used.	Strength, limitations and perspectives (p.37-38)
	23d	Discuss implications of the results for practice, policy, and future research.	Strength, limitations and perspectives (p.37-38)
**OTHER INFORMATION**			
**Registration and protocol**	24a	Provide registration information for the review, including register name and registration number, or state that the review was not registered.	Not assessed
	24b	Indicate where the review protocol can be accessed, or state that a protocol was not prepared.	Not assessed
	24c	Describe and explain any amendments to information provided at registration or in the protocol.	Not assessed)
**Support**	25	Describe sources of financial or non-financial support for the review, and the role of the funders or sponsors in the review.	Funding(p.39)
**Competing interests**	26	Declare any competing interests of review authors.	Conflict of interest(p.39)
**Availability of data, code and other materials**	27	Report which of the following are publicly available and where they can be found: template data collection forms; data extracted from included studies; data used for all analyses; analytic code; any other materials used in the review.	Data availability statement(p.39)

PRIMSA Abstract Checklist

Topic	No.	Item	Reported?
**TITLE**			
**Title**	1	Identify the report as a systematic review.	Yes
**BACKGROUND**			
**Objectives**	2	Provide an explicit statement of the main objective(s) or question(s) the review addresses.	Yes
**METHODS**			
**Eligibility criteria**	3	Specify the inclusion and exclusion criteria for the review.	Yes
**Information sources**	4	Specify the information sources (e.g. databases, registers) used to identify studies and the date when each was last searched.	Yes
**Risk of bias**	5	Specify the methods used to assess risk of bias in the included studies.	No
**Synthesis of results**	6	Specify the methods used to present and synthesize results.	Yes
**RESULTS**			
**Included studies**	7	Give the total number of included studies and participants and summarise relevant characteristics of studies.	Yes
**Synthesis of results**	8	Present results for main outcomes, preferably indicating the number of included studies and participants for each. If meta-analysis was done, report the summary estimate and confidence/credible interval. If comparing groups, indicate the direction of the effect (i.e. which group is favoured).	Yes
**DISCUSSION**			
**Limitations of evidence**	9	Provide a brief summary of the limitations of the evidence included in the review (e.g. study risk of bias, inconsistency and imprecision).	No
**Interpretation**	10	Provide a general interpretation of the results and important implications.	Yes
**OTHER**			
**Funding**	11	Specify the primary source of funding for the review.	No
**Registration**	12	Provide the register name and registration number.	No
